# Mathematical modeling, drying kinetics, and economic analysis of a hybrid photovoltaic thermal solar dryer for henna leaves

**DOI:** 10.1038/s41598-025-03460-3

**Published:** 2025-07-01

**Authors:** Abdallah Elshawadfy Elwakeel, Awad Ali Tayoush Oraiath, Tarek Hussien M. Ghanem, Ahmed Elbeltagi, Ali Salem, Ahmed Z. Dewidar, Abdelaziz M. Okasha, Abadeer Habib, Tamer M. El-Messery, Moustapha Eid Moustapha, Khaled A. Metwally

**Affiliations:** 1 Agricultural Engineering Department, Faculty of Agriculture and Natural Resources, Aswan University, Aswan, 81528 Egypt; 2 Department of Agricultural Engineering, Faculty of Agriculture, Omar Al Mukhtar University, P.O. Box 991, Al Bayda, Libya; 3 Agricultural Products Processing Engineering Department, Faculty of Agricultural Engineering, Al Azhar University, Cairo, Egypt; 4 Agricultural Engineering Department, Faculty of Agriculture, Mansoura University, Mansoura, 35516 Egypt; 5 Civil Engineering Department, Faculty of Engineering, Minia University, Minia 61111, Egypt; 6 Structural Diagnostics and Analysis Research Group, Faculty of Engineering and Information Technology, University of Pécs, Pécs 7622, Hungary; 7 Prince Sultan Bin Abdulaziz International Prize for Water Chair, Prince Sultan Institute for Environmental, Water and Desert Research, King Saud University, 11451 Riyadh, Saudi Arabia; 8 Department of Agricultural Engineering, College of Food and Agriculture Sciences, King Saud University, 11451 Riyadh, Saudi Arabia; 9 Department of Agricultural Engineering, Faculty of Agriculture, Kafrelsheikh University, Kafr El-Sheikh, 33516 Egypt; 10 International Research Centre “Biotechnologies of the Third Millennium”, Faculty of Biotechnologies (BioTech), ITMO University, St. Petersburg, Russia 191002; 11 Department of Chemistry, College of Science and Humanities, Prince Sattam Bin Abdulaziz University, 11942 Al-Kharj, Saudi Arabia; 12 Soil and Water Sciences Department, Faculty of Technology and Development, Zagazig University, Zagazig, 44519 Egypt

**Keywords:** Medical plants, Renewable energy, Drying kinetics, Thin layer modeling, Drying characteristics, Plant sciences, Environmental sciences, Energy harvesting

## Abstract

Solar dryers offer a sustainable and efficient method for drying many agricultural products, preserving their quality, color, and medicinal properties while minimizing energy consumption and environmental impact. Current research on henna processing reveals a significant gap in drying engineering studies, creating a critical barrier to process optimization and quality enhancement. While numerous studies have investigated henna’s physical and chemical characteristics, the engineering aspects of drying—including heat and mass transfer mechanisms, equipment design, and process parameter optimization—remain substantially understudied. This knowledge gap hinders the development of efficient, standardized drying methods that could improve product quality, reduce energy consumption, and increase production yields in commercial henna processing. So, during the present study, a direct solar dryer integrated with a photovoltaic system was used for drying Henna leaves at Aswan University, Egypt, during January 2025. Where a comparison study was conducted between the drying of Henna leaves by the developed direct solar dryer (DDSD) and open-air drying (OAD) at three-layer thicknesses of 2 cm, 4 cm, and 6 cm. The comparison study between both drying systems was established in terms of mathematical modeling, drying parameters, EMD, and economic analysis. The obtained results showed that the equilibrium moisture contents of Henna leaves samples dried in OAD and (DDSD) reach ranged between 2.52 and 3.23% (2.17 and 2.69%) on a dry base. Applying the DDSD to dry Henna leaves resulted in a reduction in drying time by approximately 7.14%, 13.33%, and 18.75% for layer thicknesses of approximately 2, 4, and 6 cm, respectively. Additionally, the EMD of the Henna leaves dried using the DDSD ranged from 2.84 × 10^–9^ to 22.96 × 10^–9^ m^2^/s. Furthermore, Lewis (Newton), Weibullian, and Page were the most appropriate mathematical drying models for Henna leaves at layer thicknesses of approximately 2, 4, and 6 cm, respectively, for dried samples by DDSD. On the other hand, the economic analysis revealed that the DDSD has the potential to generate substantial cost savings, amounting to 3,348 USD per year. Additionally, the payback period was calculated to be 0.077 years (less than one month), demonstrating the system’s rapid return on investment and economic viability.

## Introduction

Henna (*Lawsonia inermis*) is a powdered plant material traditionally used for medicinal and cosmetic purposes in Asia and the Mediterranean region. In North America and Europe, henna is primarily utilized for coloring hair and creating temporary body art, serving mainly cosmetic and decorative purposes rather than traditional or medicinal uses. This coloring process is due to the action of the secondary metabolite lawsone, which enables Henna to produce orange to red shades of color^1^. Where Henna powder is popular among people looking for sustainable and environmentally friendly cosmetic products because of its natural, chemical-free characteristics. Market expansion is being driven by rising demand for natural and organic products, more awareness of dangerous chemicals in conventional dyes, and the growing popularity of traditional beauty rituals across worldwide cultures^2^. The global Henna powder market size will be USD 256.5 million in 2024. Rising demand for natural hair care products is expected to boost sales to USD 391 million by 2031, with a compound annual growth rate (CAGR) of 6.20% from 2024 to 2031. Middle East and Africa had a market share of around 2% of the global revenue and was estimated at a market size of USD 5.13 million in 2024 and will grow at CAGR of 5.9% from 2024 to 2031. Additionally, Egypt Henna powder sales revenue 2024 was USD 0.54 million^3^.

When Henna leaves are dried in the traditional way, it can take two days in direct sunlight with open air and three days in the shade^4^. This drying method has numerous disadvantages, including degradation from wind-blown debris, rain, insect infestation, and intervention from humans and animals, which can lead to product contamination^5,6^. Furthermore, over 20% of Henna production is squandered throughout conventional harvesting and drying procedures because of the absence of advancements in agricultural, harvesting, and drying techniques for this plant^4^. Where the research on solar drying of henna contributes to society by promoting sustainable, energy-efficient drying methods, reducing post-harvest losses, and improving product quality. It supports rural livelihoods, enhances income for farmers, and minimizes environmental impact by replacing conventional drying techniques, fostering eco-friendly practices in the henna industry.

Dryers provide an effective substitute for solar drying of agricultural products, mitigating the issues associated with OAD^7–10^. Dryers can be classified as conventional or non-conventional based on their energy source. Traditional dryers depend on fossil fuels, leading to elevated operational expenses and environmental contamination. Non-conventional dryers utilize solar energy to reduce drying expenses, environmental degradation, and energy consumption^11–16^. Utilizing solar drying techniques, agricultural goods can be desiccated within enclosed facilities, thereby mitigating the challenges linked to conventional OAD^17–19^. The utilization of solar energy in the drying process diminishes reliance on fossil fuels (such as coal, gas, and oil), leading to a reduction in pollutant emissions^20^. Solar drying is regarded as a viable technique for food preservation due to its effective utilization of solar energy^21^. In comparison to conventional OAD, solar dryers significantly reduce drying duration, save product losses, and enhance product quality^22^. The use of solar energy for drying agricultural products in Aswan, Egypt, is very permissible, where in 2024, Aswan recorded the highest temperature around the world. This extreme heat provides an ideal environment for solar drying techniques, which can significantly reduce MC in crops while preserving their quality. Farmers in the region are increasingly adopting these methods to enhance their productivity and reduce reliance on traditional drying processes. Furthermore, Egypt, characterized by a substantial annual daily average solar radiation on a horizontal plane (8 kW h/m^2^ day) and an average daily sunlight duration of approximately 11 h, presents significant potential for harnessing solar energy as an effective resource for food drying^23–25^.

The development of drying systems, compliance with quality standards, and energy efficiency depend on evaluating the final product’s quality and forecasting the drying behavior of food under various conditions. Mathematical modeling is a cornerstone of drying technology, playing a crucial role in designing and operating dryers under ideal conditions. Since drying affects the physical, chemical, and quality traits of products, process control strategies often involve modeling drying kinetics^26–28^. Additionally, drying kinetics helps in understanding and quantitatively analyzing the thermal and physical factors involved in the process^29,30^. Key influences on drying kinetics include air temperature, humidity, product size, and drying duration^31–33^. Each factor must be carefully considered, as their effects can vary, making manual dryer operation highly inefficient. Thus, developing a model that incorporates multiple variables is vital for researchers. Over the years, various drying models—such as the Page, Midilli, and logistic models—have been developed and widely used to simulate food drying behavior^34–37^. Grasping the material transport mechanisms in thin-layer drying is critical, as it enables effective process simulation or scaling to fine-tune operating parameters. Studies indicate that relying solely on experimental drying methods, without integrating mathematical drying kinetics, can reduce dryer efficiency, increase costs, and compromise product quality. Therefore, a robust model is necessary for process design, optimization, energy management, and control. Applying mathematical models to determine the drying kinetics of agricultural products is indispensable^38,39^.

Many researchers developed many types of photovoltaic-thermal (PVT)-Based solar dryers for drying many types of agricultural crops such as Gupta et al.^40^ analyzed the performance of a hybrid PVT solar air dryer for drying green chilis; Tiwari et al.^41^ presented an attempt to explore the best PV technology suitable for PVT solar drying system through environmental and economic feasibility; Bayrak et al.^42^ analyzed a novel PVT dryer using a sustainable control approach for drying apple slices; Veeramanipriya, and Sundari^43^ evaluated the performance of hybrid PVT solar dryer for drying of cassava; Tiwari et al. ^44^ evaluated the performance of PVT mixed mode greenhouse solar dryer; Elwakeel et al.^45^ designed and implemented of a PVT-integrated solar dryer based on IoT and date fruit quality monitoring and control; and Elwakeel et al.^32^ studied the drying kinetics and thermo-environmental analysis of a PVT-operated tracking indirect solar dryer for tomato slices.

Most researches on Henna are oriented towards its chemical composition^1,46,47^, its anti-bacterial, anti-inflammatory^48,49^, antipyretic and analgesic effects^48^, while only some researches on Henna are oriented towards drying process, such as, Bennamouna et al.^50^ studied the effect of forced convective solar drying on the biological activities, Bennamouna et al.^51^ explored solar drying of Henna and its effect on the bioactivities, Bennaceur et al.^52^ studied the effect of ultrasound on Henna leaves drying and extraction of lawsone. However, no research has yet examined how solar and natural drying affect drying characteristics, EMD, drying kinetics, and the economics of the drying process. This gap in literature highlights the need for comprehensive studies that explore these variables in detail. Understanding the interplay between drying methods and their economic implications could lead to more efficient and sustainable practices in various agricultural sectors.

So, this research paper aims to investigate the mathematical modeling, drying kinetics, EMD, and economic analysis of a hybrid photovoltaic-thermal (PVT)-based direct solar dryer for Henna leaves and compare the obtained data with traditional drying methods in open air. To determine whether the DDSD offers significant advantages over open sun drying in terms of mathematical modeling, drying parameters, effective moisture diffusivity, and economic analysis to identify the optimal layer thickness for drying Henna leaves. This study will furnish pertinent data to identify the optimal drying model that can improve the drying process of Henna leaves on a commercial basis. The results of this study will benefit specialists in postharvest technology seeking to enhance the efficacy of their drying techniques. Furthermore, the findings may also contribute to the development of industry standards that ensure consistent quality in the final product. By optimizing the drying process, producers can achieve higher yields and greater marketability for Henna leaves.

## Materials and methods

### Experimental setup

**Fig. 1:**
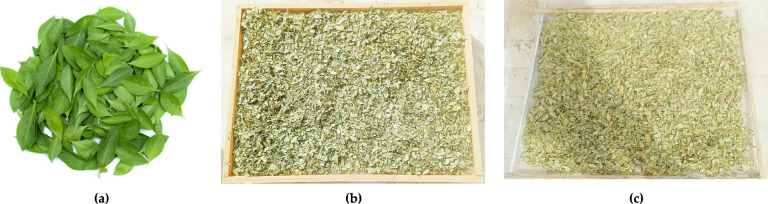
Fresh and dried Henna leaves. Whereas (**a**) fresh Henna leaves, (**b**) solar dried Henna leaves using the DDSD, and (**c**) open air-dried Henna leaves.

Henna leaves were cultivated at the research farm of Aswan University in Aswan, Egypt. Post-harvest, the Henna leaves were conveyed from the research farm to the Faculty of Agriculture and Natural Resources at Aswan University under average temperature 25 °C. Subsequently, Henna leaves samples were preserved in plastic bags at 5 ± 1 °C until the drying trials, Then, the fresh Henna leaves were distributed at three-layer thicknesses of 2, 4, and 6 cm as reported in previous studies^53,54^. Where during the current study two different drying methods were used for drying Henna leaves, the first system is a DDSD, and the other one is OAD. All field tests related to drying of fresh Henna leaves were conducted at the roof of Faculty of Agriculture and Natural Resources, Aswan University, Egypt, during January 2025. Figure 1 shows fresh and dried Henna leaves. All raw data are comprehensively presented in the Supplementary Materials, spanning Tables S1 through S5.

### Description of the developed direct solar dryer (DDSD)

**Fig. 2:**
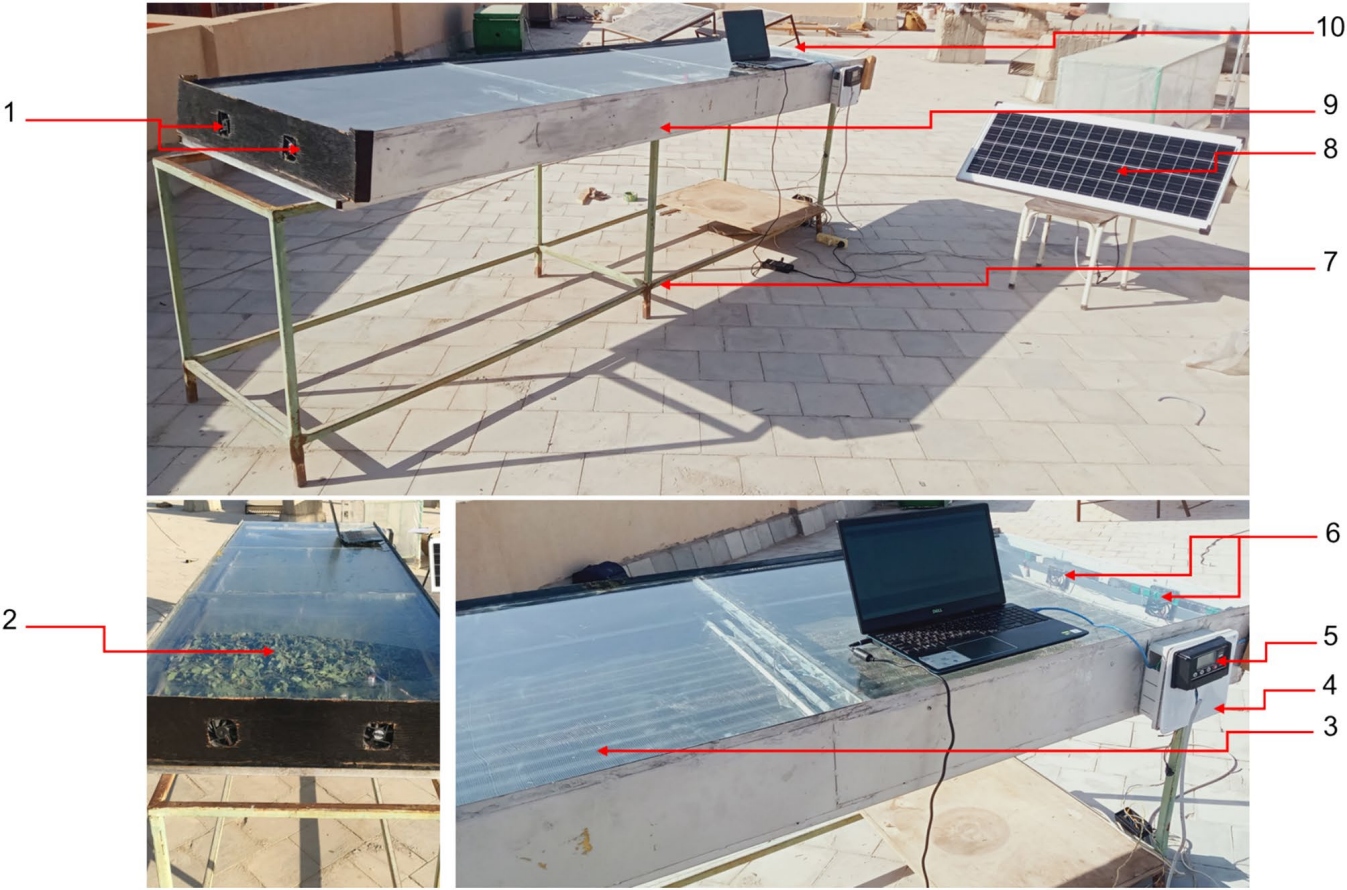
Main components of the DDSD. Whereas (1) two DC air exhaust fans, (2) Henna leaves, (3) glass cover, (4) measuring and data logger unit, (5) volt regulator, (6) two DC air suction fans, (7) metallic frame, (8) PV panel, (9) direct solar dryer, and (10) laptop.

**Fig. 3:**
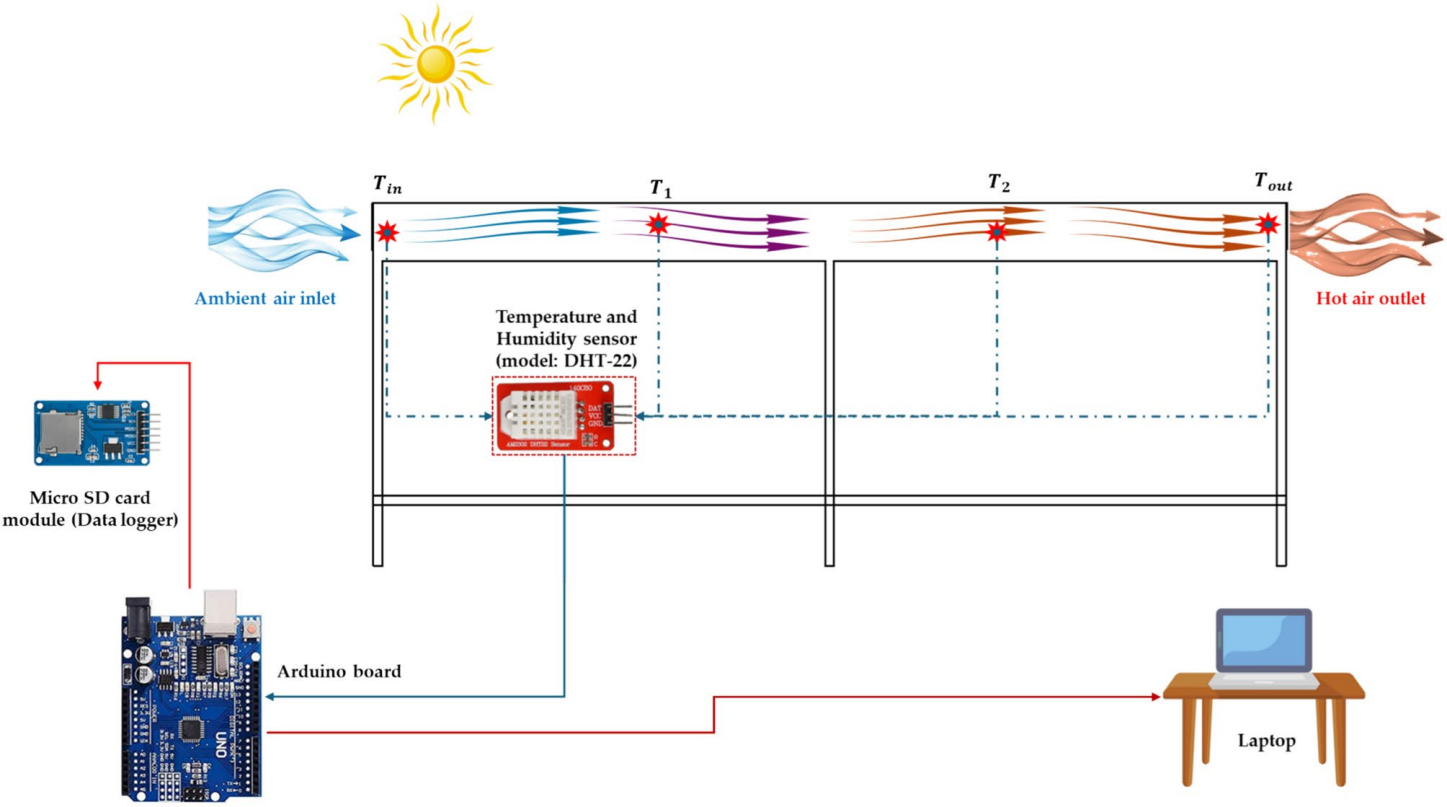
Schematic diagram showing the position of DHT sensors within the ADSD and control circuit.

The direct solar dryer was developed and used to achieve the current study’s aims. As shown in Fig. 2, the DDSD consists of many components such as, (1) two DC air exhaust fans, (2) Henna leaves, (3) glass cover, (4) measuring and data logger unit, (5) volt regulator, (6) two DC air suction fans, (7) metallic frame, (8) 100 W PV panel, (9) direct solar dryer, and (10) laptop. The DDSD has dimensions of 300 cm in length and 100 cm in width and 20 cm in depth. It was covered with a glass cover with a 3 mm in thickness. The absorber plate is made from corrugated black aluminum plate and insulated with thermal wool 3 cm in thickness. The DDSD accomplishes three drying trays, each of them has a dimension of 100 cm in length and 100 cm in width. And it was integrated with four DC brushless fan (12 V and 0.20 A, China), to distribute the air evenly around the Henna leaves to create a homogeneous MC throughout the drying trays. Where two DC fans were used to force the ambient air to the DDSD, while the other DC fans were used to exhaust the hot air from the DDSD to the outside. The DDSD was integrated with an IoT-based measuring circuit for measuring both air temperature and relative humidity of air inside and outside the DDSD. The measuring unit consists of an Arduino board (model: Uno, China), dry temperature and humidity sensor (model: DHT-22, China), micro-SD card reader module (data logger, China). The DDSD was operated with a photovoltaic (PV) system, as shown in Fig. 2. The PV system consists of a PV panel (100 W, India), and a voltage converter (12–5 V, China). Figure 3 illustrates the schematic diagram showing the position of DHT sensors within the ADSD and control circuit.

**Fig. 4:**
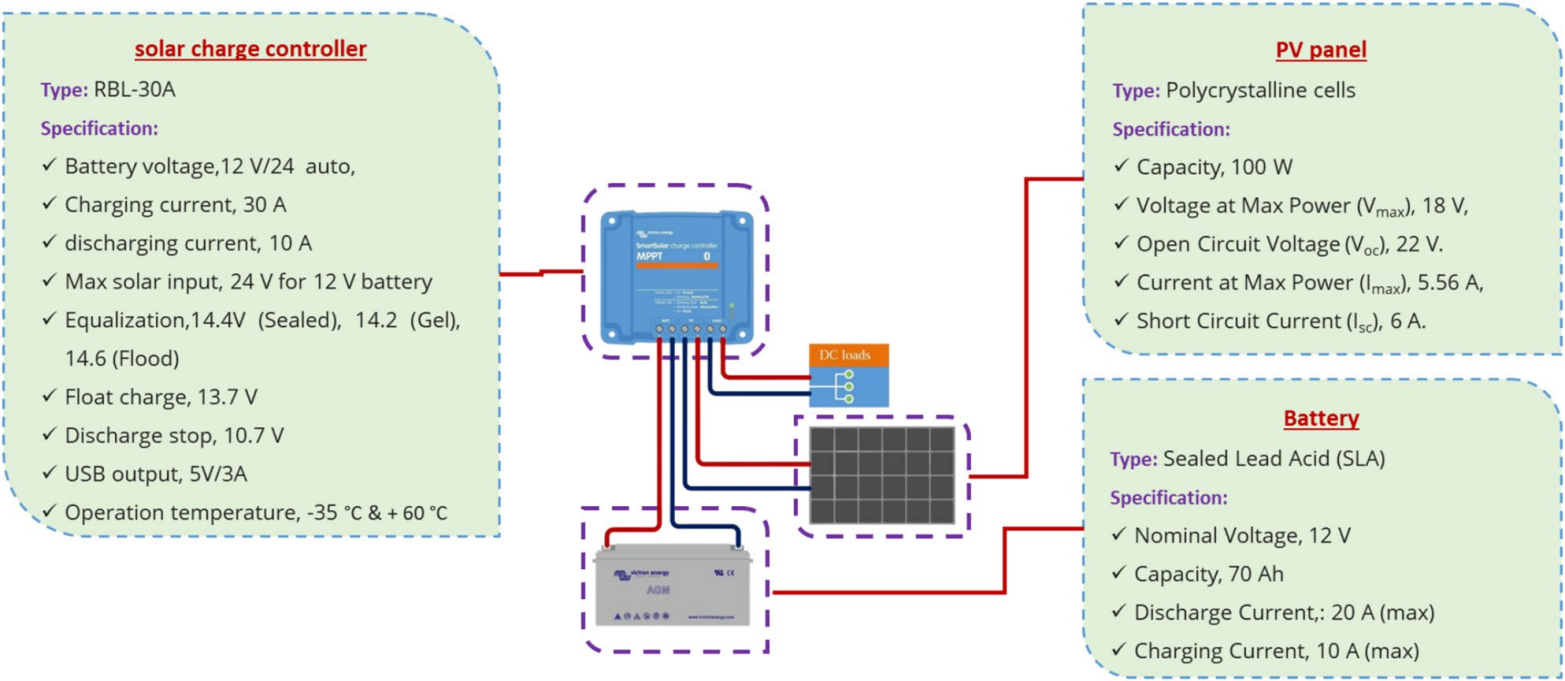
The technical specification of the PV system for the current study.

This PV system showing in Fig. 4, consisting of a 100 W polycrystalline solar panel, a 30 A solar charge controller (RBL-30A), and a 12 V/70 Ah sealed lead-acid battery, was effectively used to power an air exhaust fan and other electronic components in a solar dryer setup. The solar panel, with its maximum power voltage of 18 V and current of 5.56A, generated sufficient electricity to charge the battery through the charge controller, which regulated the charging process with specific voltage setpoints (14.4 V for equalization, 13.7 V for float) while protecting the system from over-discharge (cut-off at 10.7 V). The 12 V battery then supplied stable power to the exhaust fan (drawing less than the controller’s 10A discharge limit) and other low-power electronics, with the controller’s USB output (5 V/3A) potentially powering sensors or monitoring devices. The system’s robust operating temperature range (− 35 to + 60 °C) ensured reliable performance in varying environmental conditions, making it ideal for continuous solar dryer operation while maintaining energy efficiency.

### Evaluations processes

#### Drying parameters

##### Moisture content (MC)

Using the procedure outlined by AOAC^55^, the fresh Henna leaves sample was heated to 105 ± 1 °C in an electrical oven for three hours in order to determine the MC of the Henna leaves under laboratory conditions. According to Eke^56^, the initial MC on a dry basis (d.b.) was then determined using Eq. (1).

1 $$\mu_{d} = \left[ {\frac{{W_{w} - W_{d} }}{{W_{d} }}} \right] \times 100$$

where $\mu_{d}$ is the MC (d.b.); $W_{w} and W_{d}$ are the weight of fresh and dried Henna leaves sample, g. Drying rate (DR).

The drying rate is the velocity at which moisture is extracted from a Henna leaves during the drying process to achieve equilibrium moisture content (EMC) in a certain amount of time, usually quantified in mass per unit area per unit time. This parameter is essential in numerous drying methods and apparatus, affecting energy usage and product quality. Comprehendinthe drying rate facilitates process optimization by determining the optimal conditions for effective moisture elimination and ensuring that the final product adheres to specified standards. The DR was calculated using Eq. (2), as described by Etim et al.^57^.

2 $$DR = \frac{{M_{wt1} - M_{wt2} }}{\Delta t}$$

where the weight loss of henna sample (in g) was measured by subtracting the henna sample weight at the later time ($M_{wt2}$) from the henna sample weight at the earlier time ($M_{wt1}$), and $\Delta t$ is the interval time, h.

##### Moisture ratio (MR)

MR is the proportion of the MC at a certain time to the original MC of the Henna leaves sample. Consequently, it possesses no unit. The drying rate refers to the velocity at which interior moisture dissipates into the environment^9,22,32,33,58^. The MR of the dried Henna leaves samples under different drying methods and layer thicknesses was calculated according to Eq. (3), as mentioned by Rabha et al.^59^.

3 $$MR = \frac{{M_{t} - M_{e} }}{{M_{0} - M_{e} }}$$

where: $M_{0}$ is the initial MC in %, $M_{e}$ is the EMC in %, and $M_{t}$ is the MC at any time in %.

The MR was employed to examine the drying kinetics of Henna leaves using suitable mathematical models. The value of $M_{e}$ can be neglected, as it is relatively little compared to the values of $M_{t}$ and $M_{0}$. Consequently, as per Doymaz et al.^60^, the MR of Henna leaves can be expressed as depicted in Eq. (4).

4 $$MR = \frac{{M_{t} }}{{M_{0} }}$$

#### Drying constant (*k*)

The drying constant in thin layer drying is obtained from a combination of many drying transport characteristics, including mass coefficients, density, specific heat, thermal conductivity, and interface heat^61^. The drying constant is ascertained using the exponential correlation between LnMR and drying time. Moreover, the drying constant is obtained from the identical relationship for two drying methods (DDSD and OAD) with a three-layer thickness of the Henna leaves. Although drying constants are essential for thoroughly characterizing the drying kinetics of dried product^62,63^, it is imperative to account for the various transport parameters involved. The drying constant was calculated using Eq. (5).

5 $$MR = {\text{A exp}}\left( { - k \times t} \right)$$

where A is the initial dry basis moisture content, g_water_/g_dry matter_, k is the drying constant, and t is the interval time, h.

#### Effective moisture diffusivity (EMD)

Fick’s second law of diffusion relates the diffusive flux to the gradient of the concentration in absence of any chemical reactions. It postulates that the flux goes from regions of high concentration to regions of low concentration, with a magnitude that is proportional to the concentration gradient (spatial derivative), or in simplistic terms the concept that a solute will move from a region of high concentration to a region of low concentration across a concentration gradient. EMD is a measure of how efficient moisture can diffuse through a material. It’s influenced by factors like the material’s porosity, density, and the presence of moisture-absorbing components. Fick’s Second Law provides a framework for understanding and predicting moisture diffusion behavior in materials. Fick’s second law of diffusion describes how moisture concentration changes over time during drying processes. It states that the rate of change of moisture concentration at a point is proportional to the second derivative of concentration with respect to space. This law is applied in drying studies to model moisture diffusion in materials like food, agricultural products, and porous media, helping optimize drying conditions and predict drying rates^64–66^.

The calculation of EMD using Fick’s second law of diffusion relies on several key assumptions. These assumptions simplify the complex drying process and make mathematical modeling feasible. Here are the main assumptions including; (1) Moisture Diffusion is the Dominant Mechanism: Moisture transfer occurs primarily through diffusion, and other mechanisms (e.g., capillary flow, evaporation) are negligible or secondary; (2) Uniform Initial Moisture Content: The material (e.g., Henna leaves) has a uniform initial moisture distribution at the start of the drying process; (3) Isotropic Material: The material is isotropic, meaning its properties (e.g., diffusivity) are the same in all directions; (4) Constant Diffusivity: The effective moisture diffusivity (EMD) is assumed to be constant throughout the drying process, even though it may vary with moisture content and temperature in reality; (5) Negligible Shrinkage: The material does not undergo significant shrinkage or changes in shape during drying, which could otherwise affect the diffusion process; (6) One-Dimensional Moisture Transfer: Moisture transfer is assumed to occur primarily in one dimension (e.g., thickness of the material), simplifying the mathematical model; (7) Negligible External Resistance: The external resistance to mass transfer (e.g., boundary layer effects) is negligible compared to the internal resistance within the material; (8) Constant Drying Conditions: The temperature, humidity, and airflow around the material remain constant during the drying process; (9) Equilibrium at Surface: The surface of the material is assumed to be in equilibrium with the surrounding air, meaning the surface moisture content is directly related to the ambient relative humidity; and (10) Negligible Heat Transfer Effects: The impact of heat transfer on moisture diffusion is considered negligible, or the process is assumed to be isothermal.

Also, there are many conditions for applying Fick’s Second Law: (1) The material is homogeneous and has a well-defined geometry (e.g., slab, cylinder, or sphere); (2) The drying process occurs under controlled environmental conditions (e.g., constant temperature and humidity); (3) The boundary conditions (e.g., surface moisture content) are clearly defined and consistent throughout the process. By explicitly stating these assumptions and conditions, the application of Fick’s second law for calculating EMD becomes transparent, and the limitations of the model are clearly understood. This ensures the results are interpreted correctly within the context of the study^67–73^.

By applying Fick’s Second Law and considering the specific properties of a material, researchers and engineers can predict how moisture will move through a material over time, under different conditions. Fick’s Second Law of Diffusion is a powerful tool for understanding and predicting moisture diffusion behavior in materials. By applying this principle, researchers and engineers can gain valuable insights into EMD, leading to advancements in various fields that rely on controlling moisture transport^74^, as follows:

6 $$\frac{{\partial {\text{MC}}}}{{\partial {\text{t}}}} = {\text{D}}_{{{\text{eff}}}} \times \nabla^{2} {\text{MC}}$$

Diffusion analysis typically relies on several key assumptions, including, (1) The material starts with a consistent moisture level throughout. (2) The primary barrier to moisture movement occurs within the material itself. (3) Factors like air movement and surface conditions minimally impede moisture transfer. (4) The material’s ability to allow moisture to pass through remains unchanged during the process^75^. Using Eq. (7), the EMD can be calculated,

7 $${\text{MR}} = { }\frac{8}{{{\uppi }^{2} }} \times \mathop \sum \limits_{{{\text{n}} = 1}}^{\infty } \frac{1}{{{\text{n}}^{2} }}{\text{exp}}\left( {\frac{{ - {\uppi }^{2} \times {\text{D}}_{{{\text{eff}}}} \times {\text{t}}}}{{4{\text{L}}^{2} }}} \right)$$

where ${\text{D}}_{{{\text{eff}}}}$ is the effective moisture diffusivity, m^2^/s, n is the term number, t is the time in s, and ${\text{L}}$ is the half slab thickness.

#### Mathematical modeling of drying process

Experimental data from each drying process was analyzed using eleven drying models (Table 1). Subsequently, non-linear regression analysis was conducted utilizing Microsoft Excel (version 2016) to estimate the coefficients of the provided models and statistical measures listed in Eqs. (8–10). The optimal model was identified based on the criterion of minimal root mean squared error (RMSE) values and maximal coefficient of determination (*R*^*2*^) and adjusted coefficient of determination (${R}_{adj.}^{2}$)^76–78^.

8 $$R^{2} = 1 - \frac{{\mathop \sum \nolimits_{i = 1}^{N} \left( {MR_{pre, i} - MR_{obs, i} } \right)^{2} }}{{\mathop \sum \nolimits_{i = 1}^{N} \left( {\overline{M}R_{pre} - MR_{obs, i} } \right)^{2} }}$$

9 $$R_{adj.}^{2} = 1 - \left( {1 - R^{2} } \right)*\frac{N - 1}{{N - n}}$$

10 $$RMSE = \sqrt {\frac{1}{N}\mathop \sum \limits_{i = 1}^{N} (MR_{pre, i} - MR_{obs, i} )^{2} }$$

where $MR_{obs, i}$ and $MR_{pre, i}$ are the *i*th experimental and predicted values; $\overline{M}R_{pre}$ is the average predicted values; *N* is the number of observations; *n* is the number of constants in a model^84^.

**Table 1:** List of mathematical models of Henna leaves for OAD and DDSD.

No.	Model name	Model equation*	References
1	Aghbashlo	$MR = \exp \left( { - \frac{{k_{1} t}}{{1 + k_{2} t}}} \right)$	^79,80^
2	Henderson—Pabis	$MR = a {\text{exp}}\left( { - kt} \right)$	^38,81,82^
3	Lewis (Newton)	${\text{MR}} = {\text{exp}}\left( { - {\text{kt}}} \right)$	^81^
4	Logarithmic (Asymptotic)	${\text{MR}} = {\text{a*exp}}\left( { - {\text{kt}}} \right) + c$	^38,81,82^
5	Midilli	${\text{MR}} = {\text{a*exp}}\left( { - {\text{kt}}^{n} } \right) + bt$	^38,81,82^
6	Modified Midilli I	${\text{MR}} = {\text{exp}}\left( { - {\text{kt}}^{n} } \right) + bt$	^39,83^
7	Modified Midilli II	${\text{MR}} = {\text{a*exp}}\left( { - {\text{kt}}^{n} } \right) + b$	^39^
8	Modified Page	${\text{MR}} = {\text{exp}}\left( { - \left( {{\text{kt}}} \right)^{{\text{n}}} } \right)$	^38,81,82^
9	Page	${\text{MR}} = {\text{exp}}\left( { - {\text{kt}}^{{\text{n}}} } \right)$	^38,81,82^
10	Wang-Sigh	$MR = 1 + bt + at^{2}$	^38,81,82^
11	Weibullian	${\text{MR}} = {\text{exp}}\left( { - \left( {\frac{t}{\alpha }} \right)^{\beta } } \right)$	^39,83^
12	Weibullian I	${\text{MR}} = 10^{{ - \left( {\frac{t}{\delta }} \right)^{n} }}$	^39,83^

*MR is the moisture ratio, dimensionless; k_1,_ k_2_ and k are the drying constants, h^−1^; t is the drying time, h; a, b, c, n, ɤ, β and δ are the models constants, dimensionless.

### Uncertainty analysis

Where the uncertainties in measuring instruments are estimated using Eq. (11), according to Suraparaju et al.^85^:

11 $${\mathcal{W}}_{r} = \left[ {\left( {\frac{\partial R}{{\partial x_{1} }}{\mathcal{W}}_{1} } \right)^{2} + \left( {\frac{\partial R}{{\partial x_{2} }}{\mathcal{W}}_{2} } \right)^{2} + \cdots + \left( {\frac{\partial R}{{\partial x_{3} }}{\mathcal{W}}_{3} } \right)^{2} } \right]^{1/2}$$

The initially evaluated uncertainty of moisture content (MR), moisture ratio (MR) and drying rate (DR) was about 1.12%. The research also examined measurement errors for temperature, humidity, wind speed, and solar radiation, which were 0.32%, 0.28%, 0.24%, and 0.13%, respectively. Upon evaluating these factors, the comprehensive assessment of solar dryer efficiency was estimated to possess a cumulative uncertainty of around ± 2%. This signifies that, notwithstanding the intrinsic measurement uncertainties, the sun drying system continues to be dependable and efficient in its functioning. The accumulation of imprecision highlights the system’s resilience in practical scenarios.

### Economic analysis

Economic analysis plays a crucial role in assessing solar dryers, as it determines whether a proposed system is financially viable, cost-efficient, sustainable, and worthy of investment. For the DDSD, the economic feasibility was evaluated using Eqs. (12–23). Table 2 outlines the key assumptions used in the economic analysis, based on the Egyptian economic context.

**Table 2:** Calculation assumptions of economic analysis of the DDSD.

Parameter	Nomenclature	Unit	Value	References
Interest rate	$d$	%	20%	–
Maintenance cost	$C_{m}$	USD/year	3% of the annual capital cost	^86^
Salvage value	$V_{a}$	%	8% of the annual capital cost	^87,88^
Operating life	*τ*	year	30 years for the SD and 20 years for the PV system	^89,90^
Inflation rate	$i$	%	39.7%	–

The annual investment cost ($C_{a}$ in USD/year) of the DDSD was calculated using Eq. (12).

12 $$C_{a} = C_{ac} + C_{m} - V_{a}$$

where the annual capital cost ($C_{ac}$) of the DDSD was calculated according to Eqs. (13) and (14), as follows,

13 $$C_{ac} = C_{cc} \times F_{c}$$

14 $$F_{c} = \frac{{d\left( {1 + d} \right)^{\tau } }}{{\left( {1 + d} \right)^{\tau } - 1}}$$

where $C_{cc}$ is the capital cost of the DDSD, $F_{c}$ is the recovery factor.

The drying cost per kg of Henna leaves using the DDSD ($C_{s}$), was calculated using Eq. (15), according to Mohammed et al.^91^ and Singh et al.^91,92^.

15 $$C_{s} = \frac{{C_{a} }}{{M_{y} }}$$

The amount of dried Henna leaves using the DDSD per year ($M_{y}$) is calculated using Eq. (16), where the amount of Henna leaves using the DDSD per batch ($M_{d}$) was equal 3 kg, 6 kg, and 9 kg at layer thicknesses of 2 cm, 4 cm, and 6 cm, respectively. Additionally, the number of days available for drying per year ($D_{d}$) assumed to be 300 days.

16 $$M_{y} = \frac{{M_{d} \times D}}{{D_{d} }}$$

Based on local markets in Egypt, the cost of fresh Henna leaves ($C_{fd}$) was about 2.0 USD per kg. And the cost of one kilogram of the dried Henna leaves was calculated using Eq. (17), as recommended by Mohammed et al.^91^ and Singh et al.^91,92^.

17 $$C_{ds} = C_{dp} + C_{s}$$

where $C_{dp}$ is the cost of fresh Henna leaves per kg of dried product, which is calculated using Eq. (18),

18 $$C_{dp} = C_{fd} \times \frac{{M_{f} }}{{M_{d} }}$$

where $M_{f}$ is the quantity of fresh Henna leaves loaded inside the DDSD.

Based on local markets in Egypt, the selling price of dried Henna ($SP_{c}$) is about 7.2 USD per kg. Also, the cost savings obtained per kg of dried Henna leaves ($S_{kg}$) are given by Eq. (19),

19 $$S_{kg} = SP_{c} - C_{ds}$$

The savings obtained from the DDSD per batch ($S_{b}$) are given by Eq. 20,

20 $$S_{b} = S_{kg} \times M_{d}$$

While the savings obtained from the DDSD per day ($S_{d}$) are given by Eq. 21,

21 $$S_{d} = \frac{{S_{b} }}{D}$$

The savings obtained from the DDSD after “j” number of years is given by Eq. (22),

22 $$S_{j} = S_{d} \times D \times \left( {1 + j} \right)^{j - 1}$$

The payback time (Ŧ) for the DDSD is calculated using Eq. (23), as recommended by Mohammed et al.^91^ and Singh et al.^91,92^.

23 $${-}\!\!\!\!{\text{T}} = \frac{{ln\left[ {1 - \frac{{C_{cc} }}{{S_{1} }}\left( {d - i} \right)} \right]}}{{\ln \left( {\frac{1 + i}{{1 + d}}} \right)}}$$

where $S_{1}$ is the savings obtained from the DDSD after the first year.

## Results and discussion

**Fig. 5:**
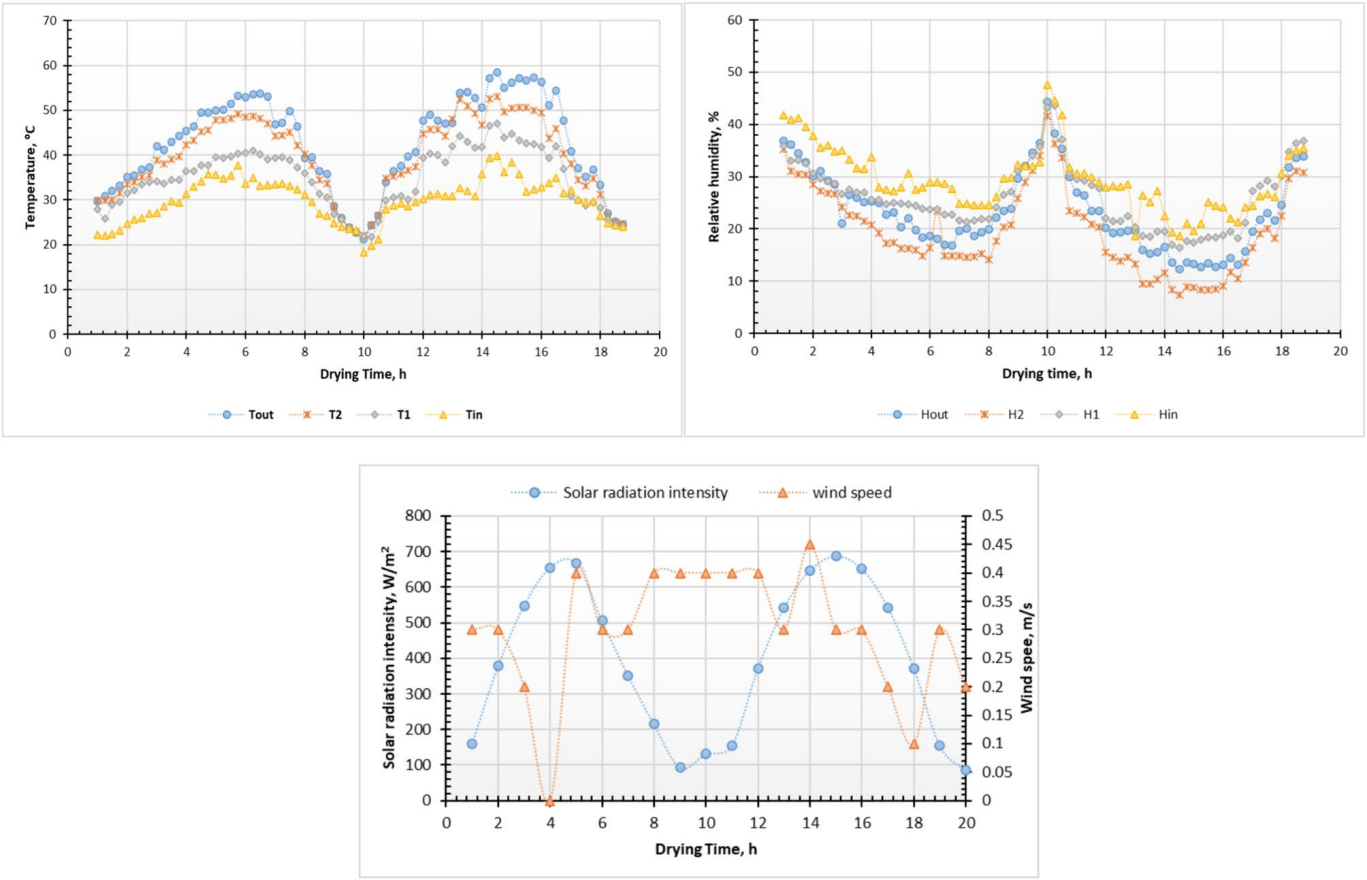
Solar radiation intensity, wind speed, temperature and humidity inside and outside the ADSD during the experiment period.

The fresh Henna leaves were dried during January 2025 at the roof of the Faculty of Agriculture and Natural Resources, Aswan University, Egypt. Where during the current study two different drying methods were used for drying Henna leaves, the first system is a direct solar dryer, and the other one is OAD at three-layer thicknesses of 2, 4, and 6 cm. Three repetitions of the MC laboratory tests revealed an average initial MC of 144.65% of fresh Henna leaves on a dry base (d.b.). During the drying process, the average ambient air temperature (in shadow), solar radiation intensity, and wind speed ranged from 10.6 to 25.72 °C, 96 to 677.5 W/m^2^, and 0.1–0.4 m/s, respectively. Also, inside the DDSD the air temperature and relative humidity ranged from 21.2 to 58.5 °C and 7.4 to 44.3%, respectively. Figure 5 shows solar radiation intensity, wind speed, temperature and humidity inside and outside the ADSD during the experiment period. Additionally, all drying experiments were conducted at fixed air velocity of 0.5 m/s.

### Effect of drying method and layer thickness on MC and drying coefficient of Henna leaves

**Fig. 6:**
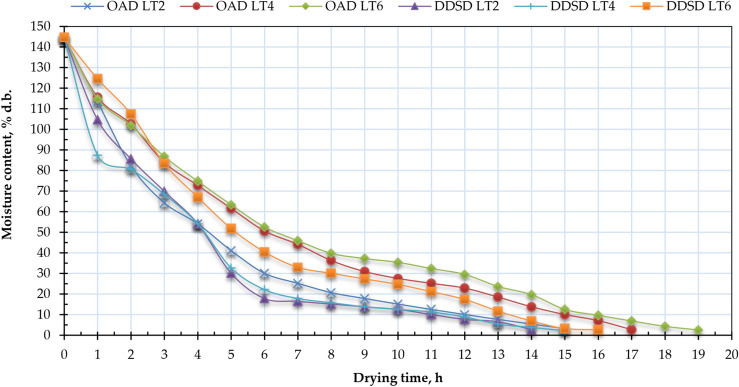
Moisture content of Henna leaves for OAD and DDSD at different layer thicknesses.

Figure 6 illustrated the impact of the drying techniques (OAD and DDSD) and layer thickness (2 cm, 4 cm, and 6 mm) on the MC of Henna leaves. Henna leaves samples dried in OAD achieved EMCs of 3.23%, 2.69%, and 2.52% (d.b.) for layer thicknesses of 2, 4, and 6 cm, respectively. While different Henna leaves samples dried by the DDSD achieved EMCs (d.b.) of 2.69%, 2.17%, and 2.69% for layer thicknesses of 2, 4, and 6 cm, respectively. The moisture extraction rate from the analyzed Henna leaves increased due to the elevated air temperature within the DDSD compared to the OAD. This results in an elevated rate of moisture evaporation from the surface layer of Henna leaves, leading to an increased drying rate^93–97^. Figure 6 illustrated that significant moisture loss transpired throughout the falling rate period, corroborating findings from numerous other investigations^98–100^.

Table 3 presents the drying coefficient (k) and determination coefficient (R^2^) for Henna leaves at various layer thicknesses, along with their respective drying methodologies. As the drying air temperature within the DDSD rises, the (k) concurrently increases relative to the OAD, as corroborated by previous studies^66,101–103^. There exists an inverse correlation between the (k) and the layer thickness of Henna leaves samples, as evidenced by the calculated (k) diminishing from 0.23 to 0.19 while the layer thickness escalated from 2 to 6 cm, as depicted in Table 3. This impact may result from a reduction in the internal temperature of the Henna leaves samples concomitant with an increase in layer thickness.

**Table 3:** Drying coefficient and determination coefficient of Henna leaves for OAD and DDSD dryer.

Coefficient	OAD			DDSD		
	LT2	LT4	LT6	LT2	LT4	LT6
k	0.23	0.19	0.181	0.252	0.249	0.228
R^2^	0.989	0.94	0.9323	0.9593	0.9742	0.9451

### Effect of drying method and layer thickness on MR of Henna leaves

**Fig. 7:**
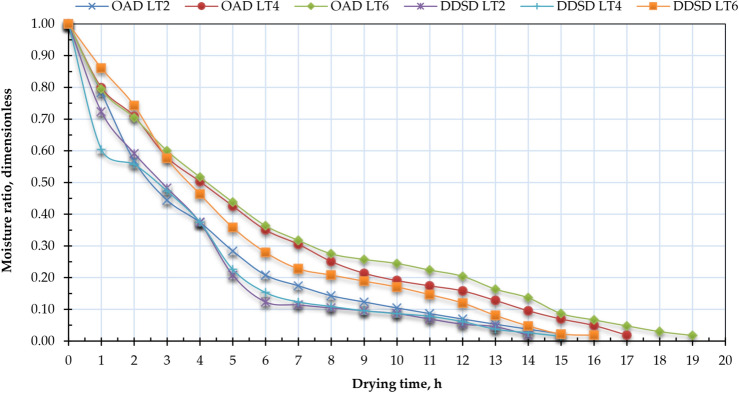
Moisture ratio of Henna leaves for OAD and DDSD at different layer thicknesses.

Figure 7 illustrates the relationship between moisture ratio (MR) and drying time for Henna leaves at three different layer thicknesses in both drying methods. Initially, the MR was high across all thickness levels. As solar radiation increased from 96 to 653 W/m^2^ during the day, the MR gradually declined. Due to the high initial moisture content in the Henna leaves and the greater thermal energy absorbed by the DDSD plate, the MR decreased faster in this system compared to open-air drying (OAD). However, as solar radiation diminished, the required drying time for moisture evaporation increased. The drying process for high-moisture agricultural products like Henna leaves typically occurs in two distinct phases. In the first stage, moisture removal is accelerated because of the high surface moisture content. Part of the thermal energy is used to evaporate surface moisture, while the rest penetrates the samples, raising their internal temperature. Under these conditions, capillary forces act between the internal water molecules, facilitating internal mass transfer, while surface moisture is simultaneously removed through external mass transfer. The reduction in the thickness of Henna leaves results in a more rapid attainment of EMC and a decreased drying duration. According to the duration required to attain the EMC, Henna leaves samples dried using the DDSD method achieve this MC after 14, 15, and 16 h for layer thicknesses of 2, 4, and 6 cm, respectively. In contrast, Henna leaves samples dried via OAD reach the EMC after 15, 17, and 19 h for layer thicknesses of 2, 4, and 6 cm, respectively. The application of DDSD for drying Henna leaves resulted in a reduction of drying time by approximately 7.14%, 13.33%, and 18.75% for layer thicknesses of 2, 4, and 6 cm, respectively.

### Effect of drying method and layer thickness on drying rate of Henna leaves

**Fig. 8:**
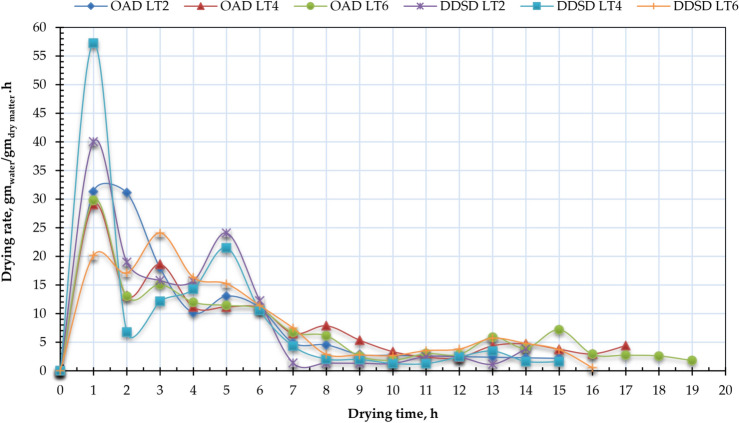
Drying rate of Henna leaves for OAD and DDSD at different layer thicknesses.

**Fig. 9:**
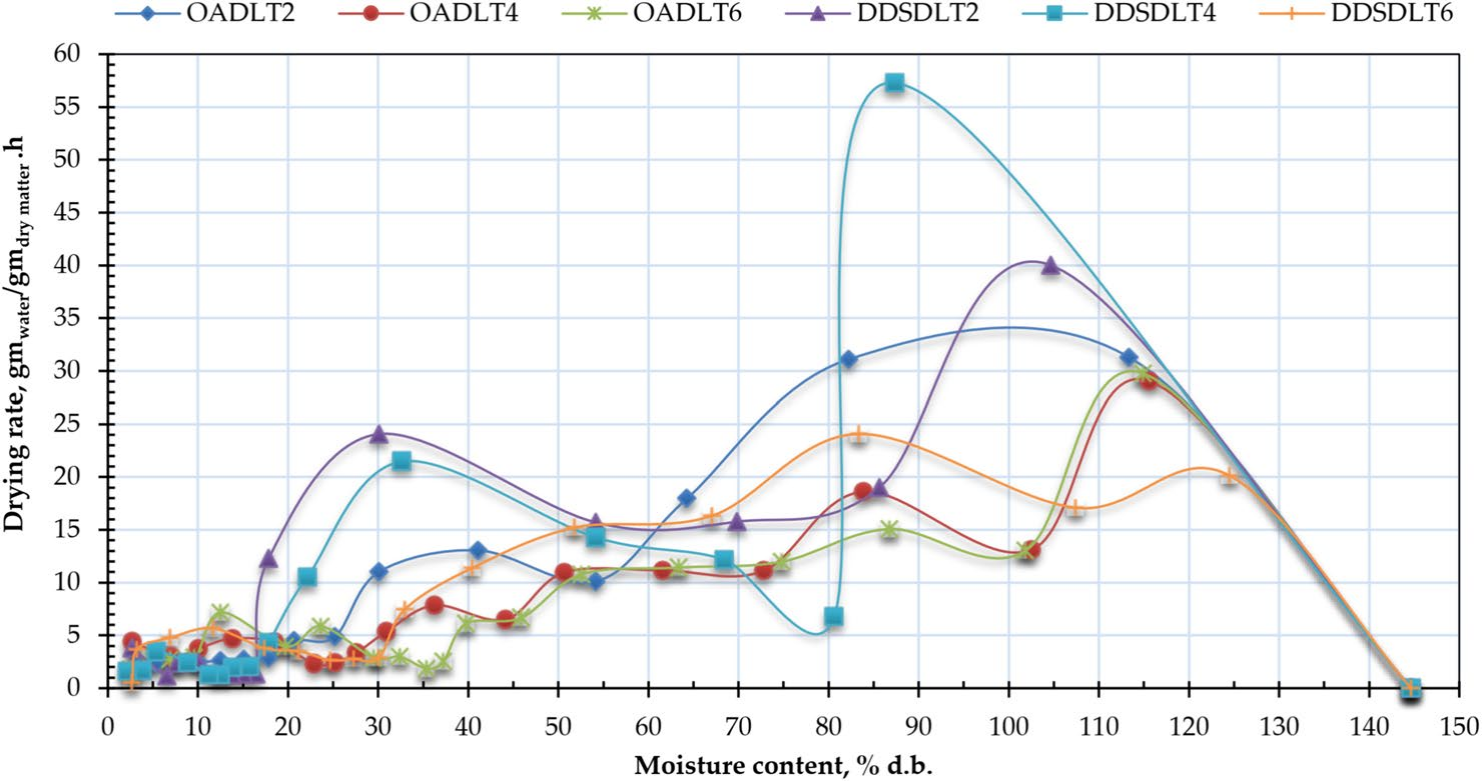
Moisture content (d.b.) vs drying rate of Henna leaves for OAD and DDSD at different layer thicknesses.

Figure 8 presents the drying rate profiles of Henna leaves for both drying methods at different layer thicknesses, while Fig. 9 shows the relationship between moisture content (MC) and drying rate (DR) across both systems. For the DDSD method, initial/maximum drying rates were 40.02, 57.28, and 20.13 kg_water_/kg_dry matter_/h at 2 cm, 4 cm, and 6 cm thicknesses respectively, compared to 31.29, 29.04, and 29.78 kg_water_/kg_dry matter_/h for open-air drying (OAD). The peak drying rate (57.28 kg_water_/kg_dry matter_/h) occurred with 4 cm layers in DDSD. High initial MC facilitates rapid surface moisture transfer and evaporation. However, as cellular water depletes, moisture migration becomes progressively slower, particularly in later stages when fiber contraction at elevated temperatures further impedes moisture diffusion. Consequently, internal diffusion emerges as the rate-limiting factor governing the overall drying process. The drying rate increases for certain thicknesses because of an optimal balance between surface evaporation, internal moisture diffusion, and heat transfer. Thicker materials often dry more slowly due to increased internal resistance, while very thin materials might dry too quickly on the surface, hindering further evaporation. These findings align with previous research, such as Elshehawy et al.^104^, who demonstrated that higher air temperatures promote faster moisture migration from a product’s core to its surface, boosting both surface evaporation and overall drying rate. Similarly, Darvishi et al.^105^ observed that the drying rate (DR) progressively declines as moisture content (MC) decreases throughout the process. The maximum DR occurred during initial drying stages when free moisture was rapidly eliminated from the Henna leaves, followed by a gradual reduction. For all tested thicknesses, drying mainly proceeded through a falling rate period, with only a brief initial constant rate phase. This pattern indicates that internal mass transfer resistance primarily governs the total drying duration. Additionally, Elshehawy et al.^104^ reported that moisture ratio (MR) reduction occurs more slowly at the experiment’s onset compared to its conclusion.

### Effect of drying method and layer thickness on EMD of Henna leaves

**Fig. 10:**
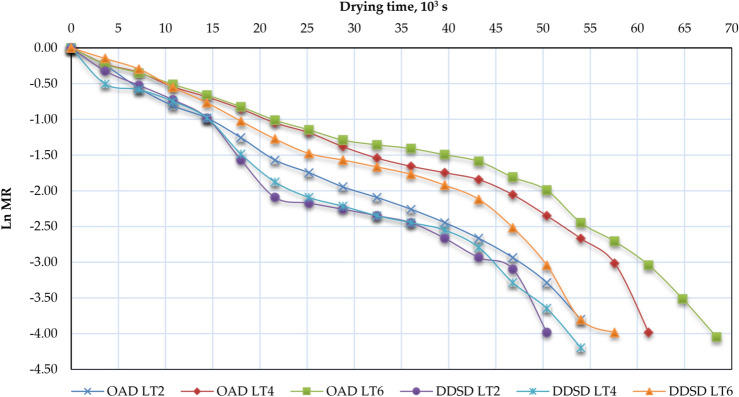
LnMR vs drying time of Henna leaves for OAD and DDSD at different layer thicknesses.

**Fig. 11:**
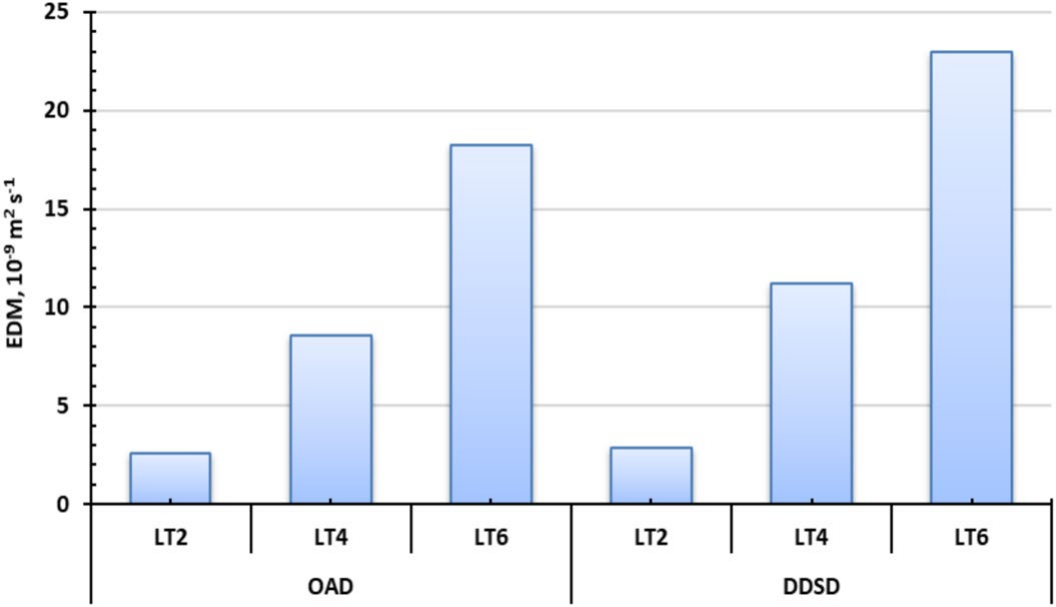
EMD of Henna leaves for OAD and DDSD at different layer thicknesses.

Figure 10 illustrates the correlation between LnMR and the drying time of Henna leaves for OAD and DDSD across various layer thicknesses. For DDSD-dried Henna leaves at 2, 4, and 6 cm layer thicknesses, EMD values ranged from 2.84 × 10^−9^ to 22.96 × 10^−9^ m^2^/s, while OSD-dried samples showed values between 2.59 × 10^−8^ and 18.22 × 10^−9^ m^2^/s (Fig. 11). As no prior studies have reported EMD values for Henna leaves, our results were compared with other agricultural products in Table 4. Notably, DDSD consistently yielded higher EMD values than OAD at equivalent thicknesses (Fig. 11). This phenomenon can be explained by temperature effects: increased air temperature accelerates water molecule movement, enhancing both diffusion rates and heat/mass transfer between the Henna leaves’ solid–liquid interface and surrounding air. The resulting reduction in surface moisture content and water vapor pressure promotes faster internal evaporation and moisture migration, ultimately increasing EMD^106^.

**Table 4:** Comparison between the obtained EMD with previous studies.

References	Drying system	Dried product	EMD, m^2^/s
Ambawat et al.^96^	Fluidized Bed Dryer	Moringa leaves	3.59–2.92 × 10^–10^
Seyedabadi^107^	Microwave	Basil leaves	1.624–7.652 × 10^–10^
López‐Ortiz et al.^108^	Solar greenhouses	Basil leaves	0.08–8.11 × 10^–10^
Mbegbu et al.^109^	Vacuum oven dryer	Scent leaves	4.76–1.74 × 10^–12^
Mbegbu et al.^109^	Vacuum oven dryer	Lemon basil leaves	4.80–2.06 × 10^–12^
Altay et al.^110^	Microwave	Purple basil	0.162–7.09 × 10^–8^
Current study	DDSD	Henna leaves	2.84–22.96 × 10^–9^

### Mathematical modeling

**Fig. 12:**
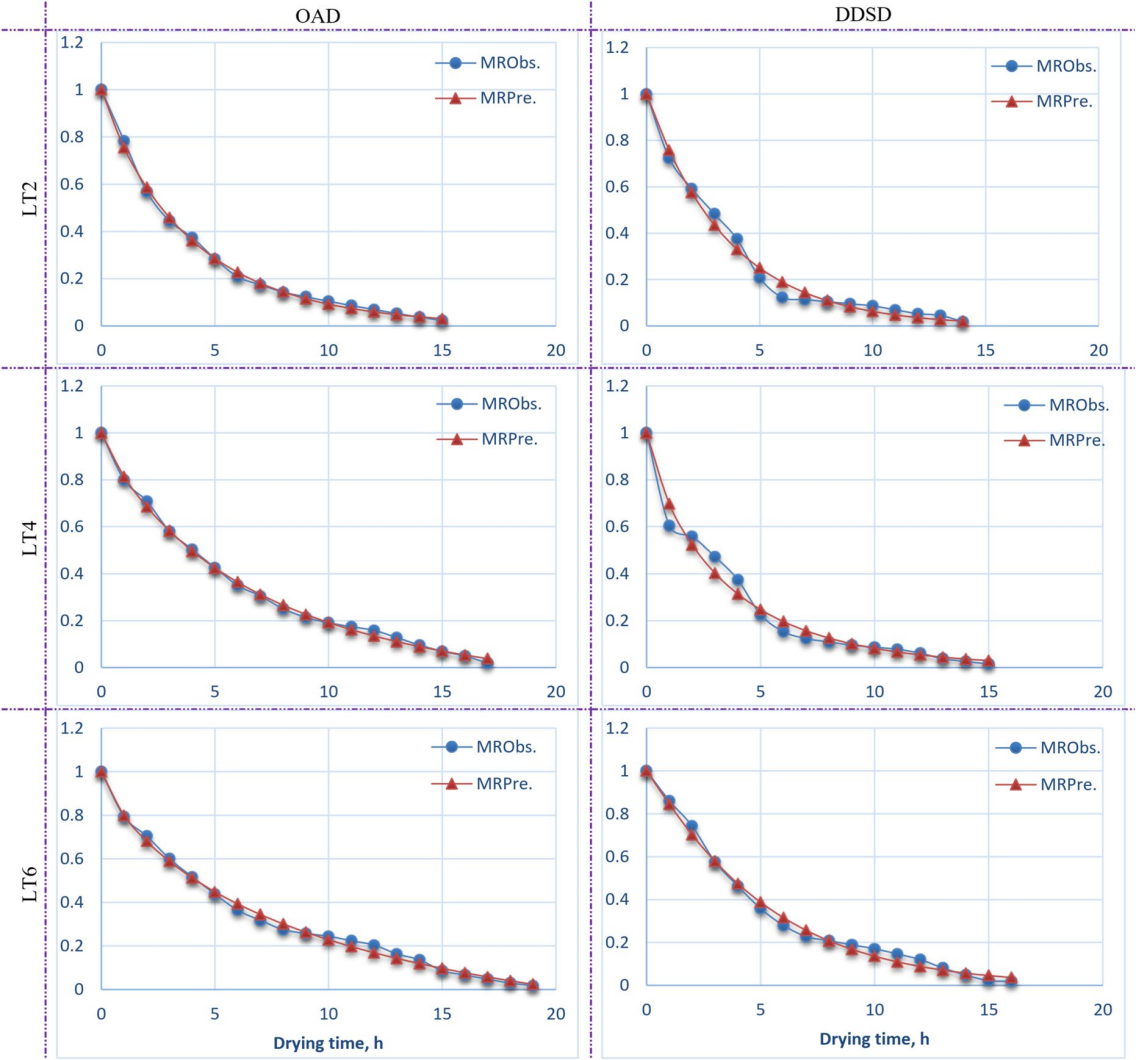
Observed and predicted MR at different drying time for the best models for both drying systems at different layer thicknesses.

Tables 5 and 6 present the constant values of mathematical models and the goodness of fit indices for Henna leaves in OAD and DDSD across various layer thicknesses. Standard computations and methodologies were employed to analyze the MC data gathered from Henna leaves samples dried using both drying processes at varying layer thicknesses. Subsequently, the MC was transformed into a MR expression, and twenty distinct drying models were employed for curve fitting calculations. The results of the statistical analysis indicate that all drying models had a good overall correlation coefficient (R^2^) (Tables 5, 6). In the present study, R^2^, R^2^_adj._, and RMSE are statistical metrics employed to evaluate the quality of the fitted models. Previous studies^76–78^ indicated that the model best appropriate for characterizing thin layer drying was the one exhibiting the highest R^2^, and R^2^_adj._, values, together with the lowest RMSE values. Several models demonstrated a strong fit to the experimental data from several samples of Henna leaves dried using DDSD and OAD techniques. The models encompassed Aghbashlo, Henderson–Pabis, Lewis (Newton), Logarithmic (Asymptotic), Midilli, Modified Midilli (I), Modified Midilli (II), Page, Modified Page, Wang-Sigh, Weibullian, and Weibullian (I). According to Table 5, among all evaluated models on the OAD, the Modified Midilli (I) was the best suitable drying model for Henna leaves at layer thicknesses of 4 cm and 6 cm, but the Weibullian (I) drying model was the most suitable for Henna leaves at a layer thickness of 2 cm. Conversely, as demonstrated in Table 6, the Lewis (Newton), Weibullian, and Page models were the most suitable drying models for Henna leaves at layer thicknesses of 2, 4, and 6 cm, respectively, for samples dried by DDSD. Figure 12 illustrates the contrast between the observed and expected MRs derived from the relevant drying models for several samples of Henna leaves subjected to both drying techniques at varying layer thicknesses.

**Table 5:** Mathematical models’ constants values and goodness of fit indices results of Henna leaves for OAD at different layer thicknesses.

MMs	LT	Models’ constants values					Goodness of fit indices		
		Parameters	Estimates	Standard error	*p* value	Sign.–Insign	RMSE	R^2^	R^2^_adj_
Aghbashlo	2	k1	0.0400	0.0252	0.1349	InSign	0.312176	− 0.100011	− 0.178584
		k1	− 0.0667	0.0223	0.0098	Sign			
	4	k1	0.1770	0.0064	6.28*10^–15^	Sign	0.018496	0.995938	0.995684
		k1	0.0040	0.0042	0.3569	InSign			
	6	k1	0.1708	0.0094	4.74*10^–13^	Sign	0.029169	0.989280	0.988684
		k1	0.0091	0.0062	0.1584	InSign			
Henderson—Pabis	2	k	0.2472	0.0056	1.79*10^–16^	Sign	0.017358	0.996599	0.996356
		a	0.9854	0.0138	2.42*10^–19^	Sign			
	4	k	0.1677	0.0034	6.01*10^–19^	Sign	0.017556	0.996340	0.996112
		a	0.9791	0.0124	3.81*10^–22^	Sign			
	6	k	0.1514	0.0047	2.57*10^–17^	Sign	0.027904	0.990190	0.989645
		a	0.9600	0.0191	8.00*10^–21^	Sign			
Lewis (Newton)	2	k	0.2509	0.0044	6.17*10^–19^	Sign	0.017407	0.996336	0.996336
	4	k	0.1714	0.0027	1.25*10^–21^	Sign	0.018467	0.995698	0.995698
	6	k	0.1581	0.0039	6.80*10^–20^	Sign	0.030238	0.987840	0.987840
Logarithmic (Asymptotic)	2	k	0.2688	0.0087	1.39*10^–13^	Sign	0.013894	0.997977	0.997665
		a	0.9722	0.0121	6.75*10^–19^	Sign			
		c	0.0255	0.0081	0.0074	Sign			
	4	k	0.1633	0.0081	2.60*10^–12^	Sign	0.017935	0.996419	0.995942
		a	0.9851	0.0165	2.89*10^–19^	Sign			
		c	− 0.0095	0.0165	0.5716	InSign			
	6	k	0.1468	0.0112	2.67*10^–10^	Sign	0.028563	0.990292	0.989149
		a	0.9665	0.0249	4.79*10^–18^	Sign			
		c	− 0.0108	0.0249	0.6695	InSign			
Midilli	2	k	0.2822	0.0160	6.17*10^–10^	Sign	0.013830	0.998149	0.997687
		a	1.0060	0.0136	2.43*10^–17^	Sign			
		b	0.0006	0.0008	0.4475	InSign			
		n	0.9394	0.0370	8.39*10^–12^	Sign			
	4	k	0.2019	0.0144	1.19*10^–9^	Sign	0.014385	0.997850	0.997390
		a	0.9995	0.0140	2.44*10^–19^	Sign			
		b	− 0.0033	0.0012	0.0180	Sign			
		n	0.8683	0.0427	8.72*10^–12^	Sign			
	6	k	0.2201	0.0210	1.40*10^–8^	Sign	0.020191	0.995434	0.994578
		a	1.0034	0.0198	4.29*10^–19^	Sign			
		b	− 0.0055	0.0017	0.0056	Sign			
		n	0.7586	0.0561	3.58*10^–10^	Sign			
Modified Midilli I	2	k	0.2775	0.0113	2.88*10^–12^	Sign	0.013396	0.998119	0.997830
		b	0.0007	0.0008	0.3847	InSign			
		n	0.9472	0.0314	2.03*10^–13^	Sign			
	4	k	**0.2023**	**0.0092**	8.40*10^**–13**^	**Sign**	**0.013898**	**0.997850**	**0.997563**
		b	− **0.0033**	**0.0012**	**0.0116**	**Sign**			
		n	**0.8674**	**0.0343**	1.02*10^**–13**^	**Sign**			
	6	k	**0.2175**	**0.0134**	8.62*10^**–12**^	**Sign**	**0.019607**	**0.995425**	**0.994887**
		b	− **0.0055**	**0.0016**	**0.0037**	**Sign**			
		n	**0.7636**	**0.0462**	6.50*10^**–12**^	**Sign**			
Modified Midilli I I	2	k	0.2835	0.0152	3.09*10^–10^	Sign	0.013643	0.998199	0.997749
		a	0.9914	0.0206	4.27*10^–15^	Sign			
		b	0.0140	0.0134	0.3182	InSign			
		n	0.9500	0.0409	2.38*10^–11^	Sign			
	4	k	0.1887	0.0111	9.07*10^–11^	Sign	0.014666	0.997765	0.997286
		a	1.0888	0.0476	1.72*10^–12^	Sign			
		b	− 0.0890	0.0415	0.0502	InSign			
		n	0.8566	0.0470	3.79*10^–11^	Sign			
	6	k	0.1857	0.0145	8.30*10^–10^	Sign	0.020711	0.995196	0.994295
		a	1.2145	0.1100	6.83*10^–9^	Sign			
		b	− 0.2107	0.1025	0.0565	InSign			
		n	0.7389	0.0622	2.38*10^–9^	Sign			
Modified Page	2	k	0.2550	0.0038	6.70*10^–19^	Sign	0.013282	0.998009	0.997866
		n	0.9267	0.0207	1.63*10^–16^	Sign			
	4	k	0.1729	0.0028	1.43*10^–20^	Sign	0.017379	0.996414	0.996190
		n	0.9552	0.0246	3.00*10^–17^	Sign			
	6	k	0.1611	0.0039	2.92*10^–19^	Sign	0.026609	0.991079	0.990584
		n	0.9097	0.0344	7.47*10^–16^	Sign			
Page	2	k	0.2819	0.0101	1.14*10^–13^	Sign	0.013282	0.998009	0.997866
		n	0.9267	0.0207	1.63*10^–16^	Sign			
	4	k	0.1870	0.0093	8.76*10^–13^	Sign	0.017379	0.996414	0.996190
		n	0.9552	0.0246	3.00*10^–17^	Sign			
	6	k	0.1900	0.0138	5.38*10^–11^	Sign	0.026609	0.991079	0.990584
		n	0.9097	0.0344	7.47*10^–16^	Sign			
Wang-Sigh	2	b	− 0.1722	0.0075	1.74*10^–12^	Sign	0.066132	0.950635	0.947109
		a	0.0076	0.0006	9.07*10^–9^	Sign			
	2	b	− 0.1291	0.0046	5.43*10^–15^	Sign	0.048802	0.971721	0.969953
		a	0.0045	0.0003	5.97*10^–10^	Sign			
	2	b	− 0.1167	0.0050	6.36*10^–15^	Sign	0.061907	0.951712	0.949030
		a	0.0036	0.0003	2.11*10^–9^	Sign			
Weibullian	2	β	0.9267	0.0207	1.63*10^–16^	Sign	0.013282	0.998009	0.997866
		α	3.9210	0.0591	6.70*10^–19^	Sign			
	4	β	0.9552	0.0246	3.00*10^–17^	Sign	0.017379	0.996414	0.996190
		α	5.7851	0.0922	1.43*10^–20^	Sign			
	6	β	0.9097	0.0344	7.47*10^–16^	Sign	0.026609	0.991079	0.990584
		α	6.2055	0.1508	2.92*10^–19^	Sign			
Weibullian I	2	n	**0.9267**	**0.0207**	1.63*10^**–16**^	**Sign**	**0.013282**	**0.998009**	**0.997866**
		δ	**9.6438**	**0.1950**	4.06*10^**–17**^	**Sign**			
	4	n	0.9552	0.0246	3.00*10^–17^	Sign	0.017379	0.996414	0.996190
		δ	13.8516	0.3280	7.70*10^–18^	Sign			
	6	n	0.9097	0.0344	7.47*10^–16^	Sign	0.026609	0.991079	0.990584
		δ	15.5227	0.5559	2.84*10^–16^	Sign			

*MMs are mathematical models: LT is the layer thickness; cm: k_1_, k_2_ and k are the drying constants, h^−1^: a, b, c, n, ɤ, β and δ are the models constants, dimensionless: RMSE is the root mean square error: R^2^ is the coefficient of determination R^2^_adj._ is the adjusted coefficient of determination at *p* ≤ 0.05.

Significant values are in bold.

**Table 6:** Mathematical models’ constants values and goodness of fit indices results of Henna leaves for DDSD at different layer thicknesses.

MMs	LT	Models’ constants values					Goodness of fit indices		
		Parameters	Estimates	Standard error	*p* value	Sign./insign	RMSE	R^2^	R^2^_adj_
Aghbashlo	2	k1	0.2766	0.0207	5.84*10^–9^	Sign	0.033593	0.988174	0.987264
		k1	− 0.0003	0.0125	0.9841	InSign			
	4	k1	0.0400	0.0277	0.1710	InSign	0.343264	− 0.422381	− 0.523980
		k1	− 0.0667	0.0245	0.0167	Sign			
	6	k1	0.0401	0.0192	0.0547	InSign	0.245140	0.384318	0.343273
		k1	− 0.0625	0.0168	0.0021	Sign			
Henderson—Pabis	2	k	0.2762	0.0124	9.59*10^–12^	Sign	0.033578	0.988185	0.987276
		a	0.9970	0.0275	1.93*10^–14^	Sign			
	4	k	0.2657	0.0161	1.43*10^–10^	Sign	0.043503	0.977155	0.975523
		a	0.9400	0.0353	2.13*10^–13^	Sign			
	6	k	0.1983	0.0061	2.45*10^–15^	Sign	0.026686	0.992704	0.992217
		a	1.0306	0.0199	2.50*10^–18^	Sign			
Lewis (Newton)	2	k	**0.2770**	**0.0095**	6.22*10^**–14**^	**Sign**	**0.032372**	**0.988174**	**0.988174**
	4	k	0.2831	0.0139	2.48*10^–12^	Sign	0.046034	0.972592	0.972592
	6	k	0.1924	0.0048	1.84*10^–17^	Sign	0.027825	0.991539	0.991539
Logarithmic (Asymptotic)	2	k	0.2841	0.0231	3.64*10^–8^	Sign	0.034671	0.988372	0.986434
		a	0.9916	0.0310	5.60*10^–13^	Sign			
		c	0.0092	0.0210	0.6691	InSign			
	4	k	0.2815	0.0297	3.31*10^–7^	Sign	0.044431	0.977871	0.974467
		a	0.9315	0.0390	3.95*10^–12^	Sign			
		c	0.0166	0.0245	0.5098	InSign			
	6	k	0.1914	0.0129	6.12*10^–10^	Sign	0.027277	0.992885	0.991869
		a	1.0385	0.0243	3.08*10^–16^	Sign			
		c	− 0.0129	0.0221	0.5691	InSign			
Midilli	2	k	0.2472	0.0391	5.72*10^–5^	Sign	0.034850	0.989231	0.986294
		a	0.9847	0.0339	9.45*10^–12^	Sign			
		b	0.0020	0.0018	0.2947	InSign			
		n	1.0967	0.1047	4.66*10^–7^	Sign			
	4	k	0.3628	0.0564	3.24*10^–5^	Sign	0.042238	0.981541	0.976926
		a	0.9853	0.0419	2.08*10^–11^	Sign			
		b	− 0.0018	0.0028	0.5321	InSign			
		n	0.8021	0.1076	7.69*10^–6^	Sign			
	6	k	0.1737	0.0244	7.68*10^–6^	Sign	0.027386	0.993341	0.991804
		a	1.0136	0.0261	7.79*10^–15^	Sign			
		b	0.0004	0.0016	0.7988	InSign			
		n	1.0706	0.0819	7.49*10^–9^	Sign			
Modified Midilli I	2	k	0.2603	0.0282	8.42*10^–7^	Sign	0.033653	0.989045	0.987219
		b	0.0018	0.0017	0.3213	InSign			
		n	1.0692	0.0843	2.60*10^–8^	Sign			
	4	k	0.3772	0.0417	5.63*10^–7^	Sign	0.040776	0.981363	0.978496
		b	− 0.0020	0.0028	0.4716	InSign			
		n	0.7829	0.0923	1.17*10^–6^	Sign			
	6	k	0.1650	0.0159	5.79*10^–8^	Sign	0.026682	0.993192	0.992219
		b	0.0006	0.0015	0.6951	InSign			
		n	1.0943	0.0662	1.39*10^–10^	Sign			
Modified Midilli I I	2	k	0.2506	0.0399	5.98*10^–5^	Sign	0.034977	0.989152	0.986194
		a	0.9565	0.0453	3.02*10^–10^	Sign			
		b	0.0276	0.0247	0.2879	InSign			
		n	1.1100	0.1176	1.32*10^–6^	Sign			
	4	k	0.3527	0.0472	7.52*10^–6^	Sign	0.041913	0.981824	0.977280
		a	1.0315	0.0847	4.09*10^–8^	Sign			
		b	− 0.0445	0.0673	0.5210	InSign			
		n	0.7782	0.1188	2.73*10^–5^	Sign			
	6	k	0.1720	0.0234	5.63*10^–6^	Sign	0.027200	0.993431	0.991915
		a	0.9963	0.0432	6.23*10^–12^	Sign			
		b	0.0158	0.0285	0.5901	InSign			
		n	1.0911	0.0904	1.94*10^–8^	Sign			
Modified Page	2	k	0.2765	0.0104	9.92*10^–13^	Sign	0.033567	0.988193	0.987285
		n	1.0090	0.0596	3.07*10^–10^	Sign			
	4	k	0.2979	0.0158	2.30*10^–11^	Sign	0.040216	0.980477	0.979082
		n	0.8406	0.0601	1.27*10^–9^	Sign			
	6	k	0.1903	0.0044	3.64*10^–17^	Sign	0.025911	0.993121	0.992663
		n	1.0750	0.0425	1.02*10^–13^	Sign			
Page	2	k	0.2733	0.0263	1.13*10^–7^	Sign	0.033567	0.988193	0.987285
		n	1.0090	0.0596	3.07*10^–10^	Sign			
	4	k	0.3613	0.0363	1.00*10^–7^	Sign	0.040216	0.980477	0.979082
		n	0.8406	0.0601	1.27*10^–9^	Sign			
	6	k	**0.1680**	**0.0135**	2.76*10^**–9**^	**Sign**	**0.025911**	**0.993121**	**0.992663**
		n	**1.0750**	**0.0425**	1.02*10^**–13**^	**Sign**			
Wang-Sigh	2	b	− 0.1895	0.0079	3.69*10^–12^	Sign	0.062592	0.958944	0.955786
		a	0.0090	0.0007	9.18*10^–9^	Sign			
	2	b	− 0.1823	0.0101	4.27*10^–11^	Sign	0.088616	0.905206	0.898435
		a	0.0083	0.0008	1.19*10^–7^	Sign			
	2	b	− 0.1419	0.0050	1.65*10^–14^	Sign	0.047803	0.976588	0.975027
		a	0.0053	0.0004	7.55*10^–10^	Sign			
Weibullian	2	β	1.0090	0.0596	3.07*10^–10^	Sign	0.033567	0.988193	0.987285
		α	3.6166	0.1357	9.92*10^–13^	Sign			
	4	β	**0.8406**	**0.0601**	1.27*10^**–9**^	**Sign**	**0.040216**	**0.980477**	**0.979082**
		α	**3.3572**	**0.1775**	2.30*10^**–11**^	**Sign**			
	6	β	1.0750	0.0425	1.02*10^–13^	Sign	0.025911	0.993121	0.992663
		α	5.2561	0.1215	3.64*10^–17^	Sign			
Weibullian I	2	n	1.0090	0.0596	3.07*10^–10^	Sign	0.033567	0.988193	0.987285
		δ	8.2657	0.4194	4.57*10^–11^	Sign			
	4	n	0.8406	0.0601	1.27*10^–9^	Sign	0.040216	0.980477	0.979082
		δ	9.0546	0.6087	5.69*10^–10^	Sign			
	6	n	1.0750	0.0425	1.02*10^–13^	Sign	0.025911	0.993121	0.992663
		δ	11.4183	0.3793	7.89*10^–15^	Sign			

*MMs are mathematical models: LT is the layer thickness; cm: k_1_, k_2_ and k are the drying constants, h^−1^: a, b, c, n, ɤ, β and δ are the models constants, dimensionless: RMSE is the root mean square error: R^2^ is the coefficient of determination R^2^_adj._ is the adjusted coefficient of determination at *p* ≤ 0.05.

Significant values are in bold.

### Economic analysis

The primary objective of the economic analysis conducted in this study was to assess the viability of combining the DDSD with a PV system for economically beneficial operation. The study utilized Eqs. (12–23), which addressed the life cycle savings technique and the simple payback methodology. Significant considerations were considered, as illustrated in Tables 7 and 8, while also evaluating the present condition of the Egyptian economy and the anticipated expenses of the DDSD components. The results indicate that using the DDSD for drying Henna leaves could yield substantial annual savings of 3348 USD. The payback period, representing the duration required to recover the initial investment, was determined to be 0.077, 0.115, and 0.228 years for layer thicknesses of 6, 4, and 2 cm, respectively. In contrast to the projected 30-year lifespan of the DDSD and the expected 20-year lifespan of the PV system. This indicates that, assuming no unexpected hazards, the DDSD will recoup its initial capital expenditure of $50.35 USD in approximately one month, demonstrating its cost-effectiveness. Table 8 presents the economic analysis of the DDSD and the PV system.

**Table 7:** Various costs related to the DDSD and the photovoltaic system.

Cost parameters	DDSD	Photovoltaic system
Capital cost, USD	200	62.5
Annual capital cost, USD	40.17	12.83
Annual maintenance cost, USD	1.205	0.385
Annual salvage value, USD	3.2135	1.027
Annualized investment cost, USD	38.16	12.19
Annual cost of the DDSD integrated photovoltaic system, USD	50.35

**Table 8:** Economic analysis of the DDSD integrated with photovoltaic system.

Economic parameters	Layer thickness		
	6 cm	4 cm	2 cm
Mass per batch, kg	9	6	3
Annually dried quantity of Henna leaves, kg/year	2700	1800	900
Cost of fresh Henna leaves, USD/kg	2.0	2.0	2.0
Selling price per, $/kg	7.2	7.2	7.2
Saving after one year, $	3348	2232	1116
Payback time, Years	0.077	0.115	0.228

## Conclusions and future work

During the current study, a comparison study between the drying of Henna leaves by the DDSD and OAD was conducted at three-layer thicknesses of 2 cm, 4 cm, and 6 cm. Where the drying process was conducted at an average initial MC of 144.65% on a dry basis. The findings showed that the EMC of Henna leaf samples dried in OAD (DSD) was 3.23% (2.69%), 2.69% (2.17%), and 2.52% (2.69%) for layer thicknesses of 2, 4, and 6 cm, in that order. Additionally, the DDSD-dried samples demonstrated a maximum drying rate of 57.28 kg_water_/kg_dry matter_/h at a 4 cm layer thickness. Using DDSD to dry Henna leaves reduced the drying time by approximately 7.14%, 13.33%, and 18.75% for layer thicknesses of 2, 4, and 6 cm, respectively. Additionally, the EMD of the dried Henna leaves dried using the DDSD ranged from 2.84 × 10^–9^ to 22.96 × 10^–9^ m^2^/s. Furthermore, Lewis (Newton), Weibullian, and Page were the most appropriate drying models for Henna leaves at layer thicknesses of 2, 4, and 6 cm, respectively, for dried samples by DDSD. Finally, the economic analysis of the DDSD showed that the annual cost of the DDSD integrated photovoltaic system is 50.35 USD; and the drying of henna leaves at a layer thickness of 6 cm led to maximizing the savings after one year to 3348 USD and minimizing the payback period to less than one month. In conclusion, drying henna leaves in a 2 cm thick layer reduced the drying time. However, the economic analysis revealed that drying henna leaves in a 6 cm thick layer was more cost-effective.

It is essential to recognize the limitations of the current study, which was conducted over a single season and focused exclusively on one crop. Furthermore, the study did not account for air speed and temperature as variables during the experimental process. Consequently, future research endeavors should prioritize the development and optimization of dryer designs, as well as their integration with hybrid renewable energy sources and exploration of potential industrial applications. Additionally, it would be beneficial to examine the effects of varying temperature and air speed levels, along with the drying of diverse products.

## Supplementary Information

Supplementary Information.

## Data Availability

All data will be made available upon request from the first author.
