# Supplementary material for: Mathematical modeling, drying kinetics, and economic analysis of a hybrid photovoltaic thermal solar dryer for henna leaves
Source: Sci Rep. 2025 Jul 1;15:21392. doi: 10.1038/s41598-025-03460-3 (PMC12215861; doi:10.1038/s41598-025-03460-3)
Supplement: Supplementary file 1 — Supplementary Information. [file 41598_2025_3460_MOESM1_ESM.docx]

**Table S1.** Weight losses for henna leaves in gm at different drying systems and layer thicknesses (LT).

| **Day** | **Time of day** | **Drying time, h** | **Open air drying (OAD)** | | | **Developed direct solar dryer (DDSD)** | | |
| --- | --- | --- | --- | --- | --- | --- | --- | --- |
|  |  |  | **LT-2 cm** | **LT-4 cm** | **LT-6 cm** | **LT-2 cm** | **LT-4 cm** | **LT-6 cm** |
| **1** | **7** | **0** | 1000.00 | 2000.00 | 3000.00 | 1000.00 | 2000.00 | 3000.00 |
|  | **8** | **1** | 898.60 | 1813.80 | 2712.60 | 864.80 | 1577.30 | 2814.00 |
|  | **9** | **2** | 763.30 | 1712.30 | 2560.30 | 780.20 | 1509.70 | 2627.80 |
|  | **10** | **3** | 661.80 | 1543.20 | 2357.40 | 695.60 | 1374.40 | 2306.60 |
|  | **11** | **4** | 594.20 | 1424.80 | 2171.20 | 594.20 | 1188.30 | 2036.20 |
|  | **12** | **5** | 492.70 | 1289.50 | 1968.20 | 391.20 | 833.10 | 1731.80 |
|  | **1** | **6** | 391.20 | 1137.30 | 1748.50 | 255.90 | 613.30 | 1460.80 |
|  | **2** | **7** | 340.50 | 1035.80 | 1596.30 | 239.00 | 511.80 | 1257.90 |
|  | **3** | **8** | 289.70 | 900.50 | 1444.00 | 222.10 | 461.00 | 1173.60 |
|  | **4** | **9** | 255.90 | 799.00 | 1376.60 | 205.20 | 410.30 | 1088.90 |
|  | **5** | **10** | 222.10 | 731.30 | 1325.80 | 188.20 | 376.50 | 1004.10 |
| **2** | **8** | **11** | 188.30 | 680.60 | 1241.10 | 154.40 | 342.70 | 886.00 |
|  | **9** | **12** | 154.40 | 629.80 | 1156.40 | 120.60 | 275.00 | 750.40 |
|  | **10** | **13** | 120.60 | 528.40 | 970.70 | 103.70 | 173.50 | 530.70 |
|  | **11** | **14** | 86.80 | 410.30 | 835.20 | 44.30 | 122.80 | 327.80 |
|  | **12** | **15** | 53.00 | 308.50 | 564.70 |  | 72.00 | 158.30 |
|  | **1** | **16** |  | 223.90 | 446.00 |  |  | 133.00 |
|  | **2** | **17** |  | 88.60 | 327.80 |  |  |  |
|  | **3** | **18** |  |  | 209.60 |  |  |  |
|  | **4** | **19** |  |  | 124.80 |  |  |  |

**Table S2.** Moisture content (w.b.) of henna leaves in gm at different drying systems and layer thicknesses (LT).

| **Day** | **Time of day** | **Drying time, h** | **Open air drying (OAD)** | | | **Developed direct solar dryer (DDSD)** | | |
| --- | --- | --- | --- | --- | --- | --- | --- | --- |
|  |  |  | **LT-2 cm** | **LT-4 cm** | **LT-6 cm** | **LT-2 cm** | **LT-4 cm** | **LT-6 cm** |
| **1** | **7** | **0** | 144.65 | 144.65 | 144.65 | 144.65 | 144.65 | 144.65 |
|  | **8** | **1** | 113.36 | 115.61 | 114.87 | 104.63 | 87.37 | 124.51 |
|  | **9** | **2** | 82.25 | 102.51 | 101.85 | 85.63 | 80.60 | 107.42 |
|  | **10** | **3** | 64.28 | 83.89 | 86.78 | 69.86 | 68.44 | 83.35 |
|  | **11** | **4** | 54.16 | 72.77 | 74.80 | 54.16 | 54.15 | 67.03 |
|  | **12** | **5** | 41.11 | 61.61 | 63.37 | 30.09 | 32.68 | 51.82 |
|  | **1** | **6** | 30.09 | 50.65 | 52.58 | 17.83 | 22.15 | 40.43 |
|  | **2** | **7** | 25.21 | 44.14 | 45.90 | 16.46 | 17.83 | 32.96 |
|  | **3** | **8** | 20.67 | 36.28 | 39.78 | 15.12 | 15.78 | 30.09 |
|  | **4** | **9** | 17.83 | 30.93 | 37.23 | 13.81 | 13.80 | 27.32 |
|  | **5** | **10** | 15.12 | 27.58 | 35.37 | 12.52 | 12.52 | 24.67 |
| **2** | **8** | **11** | 12.53 | 25.19 | 32.38 | 10.05 | 11.27 | 21.16 |
|  | **9** | **12** | 10.05 | 22.88 | 29.52 | 7.68 | 8.85 | 17.36 |
|  | **10** | **13** | 7.68 | 18.51 | 23.66 | 6.53 | 5.41 | 11.68 |
|  | **11** | **14** | 5.41 | 13.80 | 19.70 | 2.69 | 3.77 | 6.91 |
|  | **12** | **15** | 3.23 | 10.04 | 12.52 |  | 2.17 | 3.22 |
|  | **1** | **16** |  | 7.09 | 9.64 |  |  | 2.69 |
|  | **2** | **17** |  | 2.69 | 6.91 |  |  |  |
|  | **3** | **18** |  |  | 4.31 |  |  |  |
|  | **4** | **19** |  |  | 2.52 |  |  |  |

**Table S3.** Moisture ratio (MR) of henna leaves in gm at different drying systems and layer thicknesses (LT).

| **Day** | **Time of day** | **Drying time, h** | **Open air drying (OAD)** | | | **Developed direct solar dryer (DDSD)** | | |
| --- | --- | --- | --- | --- | --- | --- | --- | --- |
|  |  |  | **LT-2 cm** | **LT-4 cm** | **LT-6 cm** | **LT-2 cm** | **LT-4 cm** | **LT-6 cm** |
| **1** | **7** | **0** | 1.00 | 1.00 | 1.00 | 1.00 | 1.00 | 1.00 |
|  | **8** | **1** | 0.78 | 0.80 | 0.79 | 0.72 | 0.60 | 0.86 |
|  | **9** | **2** | 0.57 | 0.71 | 0.70 | 0.59 | 0.56 | 0.74 |
|  | **10** | **3** | 0.44 | 0.58 | 0.60 | 0.48 | 0.47 | 0.58 |
|  | **11** | **4** | 0.37 | 0.50 | 0.52 | 0.37 | 0.37 | 0.46 |
|  | **12** | **5** | 0.28 | 0.43 | 0.44 | 0.21 | 0.23 | 0.36 |
|  | **1** | **6** | 0.21 | 0.35 | 0.36 | 0.12 | 0.15 | 0.28 |
|  | **2** | **7** | 0.17 | 0.31 | 0.32 | 0.11 | 0.12 | 0.23 |
|  | **3** | **8** | 0.14 | 0.25 | 0.28 | 0.10 | 0.11 | 0.21 |
|  | **4** | **9** | 0.12 | 0.21 | 0.26 | 0.10 | 0.10 | 0.19 |
|  | **5** | **10** | 0.10 | 0.19 | 0.24 | 0.09 | 0.09 | 0.17 |
| **2** | **8** | **11** | 0.09 | 0.17 | 0.22 | 0.07 | 0.08 | 0.15 |
|  | **9** | **12** | 0.07 | 0.16 | 0.20 | 0.05 | 0.06 | 0.12 |
|  | **10** | **13** | 0.05 | 0.13 | 0.16 | 0.05 | 0.04 | 0.08 |
|  | **11** | **14** | 0.04 | 0.10 | 0.14 | 0.02 | 0.03 | 0.05 |
|  | **12** | **15** | 0.02 | 0.07 | 0.09 |  | 0.02 | 0.02 |
|  | **1** | **16** |  | 0.05 | 0.07 |  |  | 0.02 |
|  | **2** | **17** |  | 0.02 | 0.05 |  |  |  |
|  | **3** | **18** |  |  | 0.03 |  |  |  |
|  | **4** | **19** |  |  | 0.02 |  |  |  |

**Table S4.** Drying rate (**gm _water_/gm _drymatter.h_**) of henna leaves in gm at different drying systems and layer thicknesses (LT).

| **Day** | **Time of day** | **Drying time, h** | **Open air drying (OAD)** | | | **Developed direct solar dryer (DDSD)** | | |
| --- | --- | --- | --- | --- | --- | --- | --- | --- |
|  |  |  | **LT-2 cm** | **LT-4 cm** | **LT-6 cm** | **LT-2 cm** | **LT-4 cm** | **LT-6 cm** |
| **1** | **7** | **0** | 0.00 | 0.00 | 0.00 | 0.00 | 0.00 | 0.00 |
|  | **8** | **1** | 31.29 | 29.04 | 29.78 | 40.02 | 57.28 | 20.13 |
|  | **9** | **2** | 31.11 | 13.10 | 13.02 | 19.00 | 6.76 | 17.09 |
|  | **10** | **3** | 17.97 | 18.62 | 15.08 | 15.77 | 12.17 | 24.07 |
|  | **11** | **4** | 10.12 | 11.12 | 11.98 | 15.70 | 14.28 | 16.32 |
|  | **12** | **5** | 13.05 | 11.17 | 11.42 | 24.07 | 21.48 | 15.21 |
|  | **1** | **6** | 11.02 | 10.95 | 10.79 | 12.26 | 10.53 | 11.39 |
|  | **2** | **7** | 4.88 | 6.52 | 6.68 | 1.37 | 4.32 | 7.47 |
|  | **3** | **8** | 4.54 | 7.86 | 6.12 | 1.34 | 2.05 | 2.87 |
|  | **4** | **9** | 2.84 | 5.35 | 2.55 | 1.31 | 1.97 | 2.76 |
|  | **5** | **10** | 2.71 | 3.34 | 1.86 | 1.29 | 1.28 | 2.65 |
| **2** | **8** | **11** | 2.59 | 2.39 | 2.99 | 2.47 | 1.25 | 3.52 |
|  | **9** | **12** | 2.48 | 2.31 | 2.86 | 2.37 | 2.42 | 3.80 |
|  | **10** | **13** | 2.37 | 4.37 | 5.86 | 1.15 | 3.44 | 5.67 |
|  | **11** | **14** | 2.27 | 4.71 | 3.95 | 3.84 | 1.64 | 4.77 |
|  | **12** | **15** | 2.17 | 3.77 | 7.18 |  | 1.59 | 3.69 |
|  | **1** | **16** |  | 2.95 | 2.89 |  |  | 0.53 |
|  | **2** | **17** |  | 4.40 | 2.73 |  |  |  |
|  | **3** | **18** |  |  | 2.60 |  |  |  |
|  | **4** | **19** |  |  | 1.79 |  |  |  |

**Table S5.** Temperature and humidity inside and outside the ADSD during the experiment period.

| **1st day 17/1/2025** | | | | | | | | | | | | | | | |
| --- | --- | --- | --- | --- | --- | --- | --- | --- | --- | --- | --- | --- | --- | --- | --- |
| **Time of day** | **Temperature ᵒC** | | | | | | | | **Humidity %** | | | | | | |
|  | **T out** | | **T2** | | **T1** | | **Tin** | | **T out** | **T2** | | **T1** | | **Tin** | |
| **8** | 29.7 | | 29.9 | | 28 | | 22.2 | | 36.8 | 35.3 | | 35.9 | | 41.8 | |
|  | 30.8 | | 29.9 | | 25.8 | | 22.1 | | 36.1 | 31 | | 33 | | 41 | |
|  | 32 | | 30 | | 28.9 | | 22.4 | | 34.4 | 30.5 | | 33.2 | | 41.2 | |
|  | 33.1 | | 31.5 | | 29.5 | | 23.2 | | 32.8 | 30.4 | | 32.6 | | 39.5 | |
| **9** | 35.1 | | 33.5 | | 31.6 | | 24.7 | | 30.1 | 28.5 | | 30.7 | | 37.9 | |
|  | 35.5 | | 33.9 | | 32.2 | | 25.6 | | 31.1 | 27.2 | | 29.7 | | 35.6 | |
|  | 36.8 | | 35.1 | | 33.5 | | 25.9 | | 29.3 | 26.9 | | 29.1 | | 36 | |
|  | 37.2 | | 35.5 | | 33.9 | | 26.9 | | 28.5 | 26.7 | | 28.6 | | 34.9 | |
| **10** | 42 | | 38.9 | | 34.2 | | 27.1 | | 21 | 24.2 | | 26.7 | | 35 | |
|  | 41.1 | | 38.1 | | 33.6 | | 28.6 | | 26.6 | 22.6 | | 27.5 | | 33.3 | |
|  | 43 | | 39.1 | | 34.4 | | 29.7 | | 26 | 22.5 | | 27 | | 31.7 | |
|  | 44.2 | | 39.7 | | 34.4 | | 29.4 | | 25.2 | 21.5 | | 27 | | 31.5 | |
| **11** | 45.4 | | 42.3 | | 36.4 | | 31.3 | | 25.3 | 20.7 | | 25.5 | | 33.8 | |
|  | 46.3 | | 43.3 | | 36.5 | | 33 | | 25 | 19.2 | | 25.5 | | 28 | |
|  | 49.4 | | 45.3 | | 37.8 | | 34.2 | | 22.8 | 17.3 | | 24.7 | | 27.6 | |
|  | 49.5 | | 45.5 | | 37.7 | | 35.8 | | 23.1 | 17.4 | | 25 | | 27.2 | |
| **12** | 50 | | 47.9 | | 39.5 | | 35.6 | | 20.4 | 16.2 | | 24.9 | | 27.9 | |
|  | 50.2 | | 47.9 | | 39.4 | | 34.8 | | 22 | 16.3 | | 24.7 | | 30.7 | |
|  | 51.4 | | 48.1 | | 39.7 | | 35.4 | | 19.8 | 16 | | 24.5 | | 27.6 | |
|  | 53.3 | | 49.1 | | 40.4 | | 37.7 | | 18.4 | 14.9 | | 23.8 | | 27.9 | |
| **13** | 52.9 | | 48.5 | | 40.5 | | 33.7 | | 18.6 | 16.4 | | 23.7 | | 29 | |
|  | 53.5 | | 48.6 | | 41 | | 35 | | 18.1 | 23.4 | | 23.7 | | 29 | |
|  | 53.7 | | 48.1 | | 40.1 | | 33.2 | | 16.9 | 14.9 | | 22.8 | | 28.7 | |
|  | 53 | | 47 | | 39 | | 33.3 | | 16.8 | 14.8 | | 22.7 | | 27.7 | |
| **14** | 46.8 | | 44.3 | | 39.4 | | 33.4 | | 19.7 | 14.8 | | 21.6 | | 24.9 | |
|  | 47.2 | | 44.4 | | 39.5 | | 33.7 | | 20.1 | 14.6 | | 21.3 | | 24.9 | |
|  | 49.8 | | 45 | | 38.8 | | 33.2 | | 18.6 | 14.7 | | 21.6 | | 24.6 | |
|  | 46.4 | | 42.2 | | 37.2 | | 32.4 | | 19.3 | 15.2 | | 21.9 | | 24.6 | |
| **15** | 39.4 | | 40.2 | | 36 | | 31.2 | | 19.9 | 14.1 | | 21.9 | | 24.6 | |
|  | 39.5 | | 37.7 | | 33.9 | | 29.5 | | 22.2 | 17.7 | | 24.1 | | 26.1 | |
|  | 36.5 | | 34.4 | | 31.3 | | 26.9 | | 23.5 | 20.3 | | 26.6 | | 29.7 | |
|  | 35.7 | | 33.7 | | 30.6 | | 26.5 | | 23.9 | 20.8 | | 27.1 | | 29.8 | |
| **16** | 28.6 | | 28.5 | | 26.8 | | 24.8 | | 29.6 | 25.8 | | 31 | | 32.3 | |
|  | 26 | | 25.7 | | 25.6 | | 24 | | 32 | 29 | | 32 | | 32.1 | |
|  | 23.9 | | 23.9 | | 23.9 | | 23.6 | | 34.6 | 31.2 | | 34 | | 32.1 | |
|  | 22.7 | | 22.9 | | 23 | | 23.2 | | 36.4 | 34 | | 35.7 | | 32.8 | |
|  | | **2nd day 17/1/2025** | | | | | | | | | | | | | |
| **Time of day** | | **Temperature ᵒC** | | | | | | | **Humidity %** | | | | | | |
|  |  | **T out** | | **T2** | | **T1** | | **Tin** | **T out** | | **T2** | | **T1** | | **Tin** |
| **8** | | 21.2 | | 22 | | 21.9 | | 18.3 | 44.3 | | 41.7 | | 43.2 | | 47.6 |
|  |  | 24.4 | | 24.4 | | 21.7 | | 19.7 | 38.3 | | 36.3 | | 43.6 | | 44.6 |
|  |  | 26.4 | | 26.4 | | 25.2 | | 21.3 | 35.3 | | 33.6 | | 37.2 | | 41.8 |
|  |  | 33.9 | | 34.8 | | 29.9 | | 27.9 | 30 | | 23.5 | | 30.5 | | 31.8 |
| **9** | | 36.5 | | 35.3 | | 30.5 | | 28.7 | 27 | | 23 | | 29.5 | | 30.6 |
|  |  | 37.5 | | 35.7 | | 30.8 | | 29.2 | 26.4 | | 22.3 | | 29.3 | | 30.6 |
|  |  | 39.7 | | 36.6 | | 30 | | 28.6 | 23.5 | | 20.9 | | 28.4 | | 30 |
|  |  | 40.7 | | 37.4 | | 31.9 | | 29.6 | 23.5 | | 20.3 | | 27.8 | | 29 |
| **10** | | 47.7 | | 44.7 | | 39.4 | | 30.2 | 20.2 | | 15.5 | | 22.1 | | 28 |
|  |  | 49 | | 45.7 | | 40.3 | | 31.2 | 19.2 | | 14.6 | | 21.4 | | 28.3 |
|  |  | 47.7 | | 45.7 | | 40 | | 31.4 | 19.3 | | 13.9 | | 21.5 | | 28.1 |
|  |  | 47 | | 44.3 | | 38.3 | | 30.9 | 19.6 | | 14.5 | | 22.5 | | 28.5 |
| **11** | | 47.2 | | 48 | | 42 | | 30.9 | 19.6 | | 13.3 | | 20.3 | | 18.7 |
|  |  | 53.9 | | 52.4 | | 44.2 | | 32.6 | 16 | | 9.5 | | 18.7 | | 26.4 |
|  |  | 54 | | 51 | | 43 | | 32 | 15.3 | | 9.5 | | 18.5 | | 25.2 |
|  |  | 52.7 | | 49.3 | | 41.6 | | 30.8 | 15.6 | | 10.3 | | 19.5 | | 27.2 |
| **12** | | 50.6 | | 46.7 | | 41.8 | | 35.8 | 16.5 | | 11.6 | | 19.5 | | 22.5 |
|  |  | 57.2 | | 52.5 | | 46.5 | | 39.4 | 13.6 | | 8.3 | | 17 | | 19.4 |
|  |  | 58.5 | | 53 | | 47 | | 39.8 | 12.3 | | 7.4 | | 16.4 | | 18.7 |
|  |  | 55 | | 49.7 | | 44 | | 36.3 | 13.5 | | 8.9 | | 17.6 | | 21.1 |
| **13** | | 56.1 | | 50.4 | | 44.7 | | 38.3 | 13.3 | | 8.7 | | 17.4 | | 19.6 |
|  |  | 57.1 | | 50.6 | | 43.3 | | 35.7 | 12.7 | | 8.3 | | 17.9 | | 21.1 |
|  |  | 56.7 | | 50.6 | | 42.6 | | 31.9 | 13.4 | | 8.4 | | 18.4 | | 25.1 |
|  |  | 57.3 | | 50 | | 42.4 | | 32.4 | 12.7 | | 8.5 | | 18.3 | | 24.4 |
| **14** | | 56.4 | | 49.4 | | 41.8 | | 32.8 | 13.2 | | 9 | | 18.8 | | 24.2 |
|  |  | 51.1 | | 43.7 | | 39.3 | | 33.8 | 14.4 | | 11.7 | | 19.5 | | 22.1 |
|  |  | 54.4 | | 45.9 | | 42 | | 35 | 13.2 | | 10.5 | | 18.2 | | 21.3 |
|  |  | 47.7 | | 40.39 | | 36.9 | | 31.6 | 15.7 | | 13.6 | | 21.2 | | 24.2 |
| **15** | | 40.9 | | 38 | | 30.9 | | 32.2 | 19.5 | | 16.4 | | 27.2 | | 24.4 |
|  |  | 37.1 | | 34.5 | | 29.9 | | 30.1 | 21.7 | | 19 | | 28.2 | | 26.3 |
|  |  | 35.1 | | 33.2 | | 28.8 | | 29.5 | 23 | | 20.1 | | 29.2 | | 26.7 |
|  |  | 36.7 | | 34.8 | | 29.4 | | 29.7 | 21.6 | | 18.2 | | 28.1 | | 26.1 |
| **16** | | 33.3 | | 31.2 | | 28.3 | | 26.4 | 24.6 | | 22.5 | | 30.1 | | 30.7 |
|  |  | 26.9 | | 26.4 | | 25.6 | | 24.8 | 31.8 | | 29.6 | | 34.7 | | 34.1 |
|  |  | 25.1 | | 25 | | 24.4 | | 24.3 | 33.6 | | 31.1 | | 36.5 | | 35 |
|  |  | 24.5 | | 24.6 | | 24.2 | | 24 | 33.9 | | 30.8 | | 36.8 | | 35.5 |
